# A Resorcin[4]arene-Based Phosphite-Phosphine Ligand for the Branched-Selective Hydroformylation of Alkyl Alkenes

**DOI:** 10.1021/acscatal.4c03510

**Published:** 2024-07-24

**Authors:** Jennifer E. Smart, Jack Emerson-King, Rebekah J. Jeans, Thomas M. Hood, Samantha Lau, Alejandro Bara-Estaún, Ulrich Hintermair, Paul G. Pringle, Adrian B. Chaplin

**Affiliations:** † Department of Chemistry, University of Warwick, Coventry CV4 7AL, U.K.; ‡ Dynamic Reaction Monitoring Facility and Department of Chemistry, University of Bath, Bath BA2 7AY, U.K.; § School of Chemistry, University of Bristol, Bristol BS8 1TS, U.K.

**Keywords:** hydroformylation, regioselectivity, rhodium catalysts, cavitand ligands, chelating phosphine ligands

## Abstract

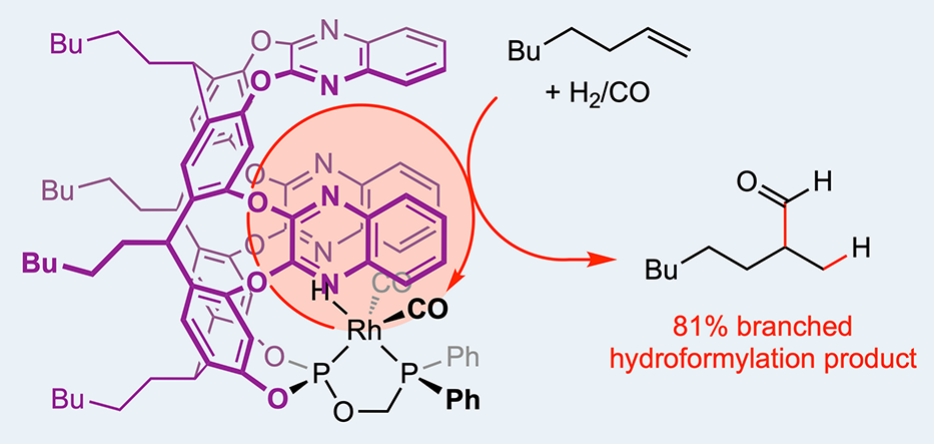

Synthesis of a chelating phosphite-phosphine ligand from a tris(quinoxaline) extended resorcin[4]arene and its application in the rhodium-catalyzed hydroformylation of terminal alkyl alkenes are reported. Rhodium complexes are formed within the cavity of the macrocycle and branched-selective hydroformylation of 1-octene with a *b*/*l* ratio of 5.9 has been achieved at 60 °C under 1:1 H_2_/CO (20 bar).

The hydroformylation of alkenes is a technologically important transformation which enables the high-volume production of aldehydes in the chemical industry.^1^ While the transition-metal-catalyzed addition of H_2_ and CO to an alkene can give both linear and branched aldehyde isomers (Figure 1A), attention has historically been devoted to the development of systems that deliver high regioselectivity for the former, which are of the greatest commercial interest for bulk chemical manufacture. Many linear-selective catalysts are known, and the hydroformylation of propylene can be carried out on an industrial scale with *l/b* > 30 using a proprietary rhodium diphosphite catalyst system.^2^ Motivated by applications in fine chemical synthesis, there is growing demand for catalysts which promote branched-selective hydroformylation, but few examples have been reported in the literature.^3^ Rhodium complexes of porphyrin-based encapsulating phosphine ligands and the chelating phosphite-phosphine ligand bobphos are state-of-the-art examples which promote the hydroformylation of terminal alkyl alkenes with *b*/*l* ratios of up to 3 at low temperature (Figure 1B).^4−7^ As part of our work with cavitand-based ditopic ligands,^8^ we reasoned that the new resorcin[4]arene-based ligand “JEKphos” would marry the key structural and electronic features of these innovative systems and a rhodium(I) complex should promote the branched selective hydroformylation of alkyl alkenes (Figure 1C).^9^ We herein report on the synthesis and coordination chemistry of JEKphos along with evaluation of this ligand in the rhodium-catalyzed hydroformylation of 1-hexene, 1-heptene, and 1-octene against bobphos and dppe benchmarks.

**Figure 1:**
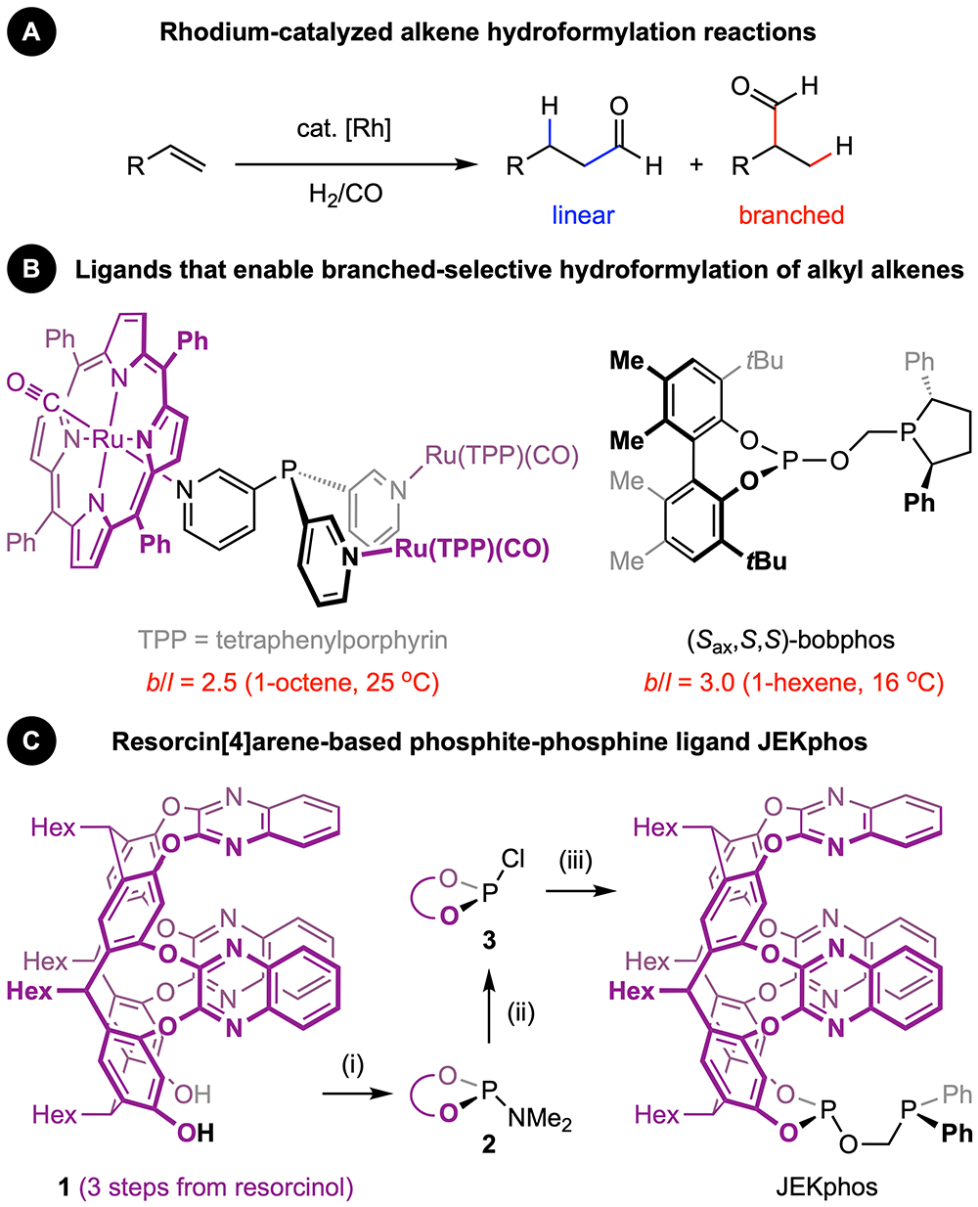
(A) Rhodium catalysed alkene hydroformylation reactions. Phosphorous-based ancillary ligands used to promote regioselective formation of branched aldehyde products (B) reported in the literature, and (C) discussed in this work. Reagents and conditions: (i) P(NMe_2_)_3_, Et_3_N, toluene, 75 °C (32% yield); (ii) HCl in Et_2_O, THF, RT (98% yield); (iii) HOCH_2_PPh_2_ from paraformaldehyde and HPPh_2_, Et_3_N, CH_2_Cl_2_, RT (88% yield).

Synthesis of JEKphos was achieved in six steps from resorcinol (Figure 1C), taking advantage of literature procedures for the preparation of tris(quinoxaline) extended resorcin[4]arene **1** and an undecyl variant of phosphoramidite **2** (δ_31P_ 142.0).^10^ Following conversion into chlorophosphite **3** (δ_31P_ 121.3), treatment with HOCH_2_PPh_2_ in the presence of Et_3_N afforded JEKphos, which was isolated as an analytically pure white solid upon workup (δ_31P_ 127.5, –14.5; ^3^*J*_PP_ = 5 Hz). Before evaluation of JEKphos in catalysis, preparation of a cationic rhodium(I) derivative was targeted to facilitate structural elucidation by single crystal X-ray diffraction. A series of complexes of the form [Rh(JEKphos)(norbornadiene)]^+^**4** (δ_31P_ 160.8, ^1^*J*_RhP_ = 263 Hz; 65.7, ^1^*J*_RhP_ = 153 Hz; ^2^*J*_PP_ = 51 Hz) partnered with different counterions were studied, with carborane-based **4**[HCB_11_Me_5_I_6_] ultimately found to be amenable to crystallographic analysis (Figure 2A). The resulting solid-state structure demonstrates binding of the rhodium(I) norbornadiene fragment within the cavity of the resorcin[4]arene and chelation of JEKphos with a bite angle of 82.16(4)°. Analysis of **4** by ^1^H NMR spectroscopy in CD_2_Cl_2_ indicates that the metal fragment remains encapsulated in solution, with the diastereotopic methylene protons of the diene that are projected into a quinoxaline wall shifted significantly upfield to δ −2.2 and −2.7.^11^

**Figure 2:**
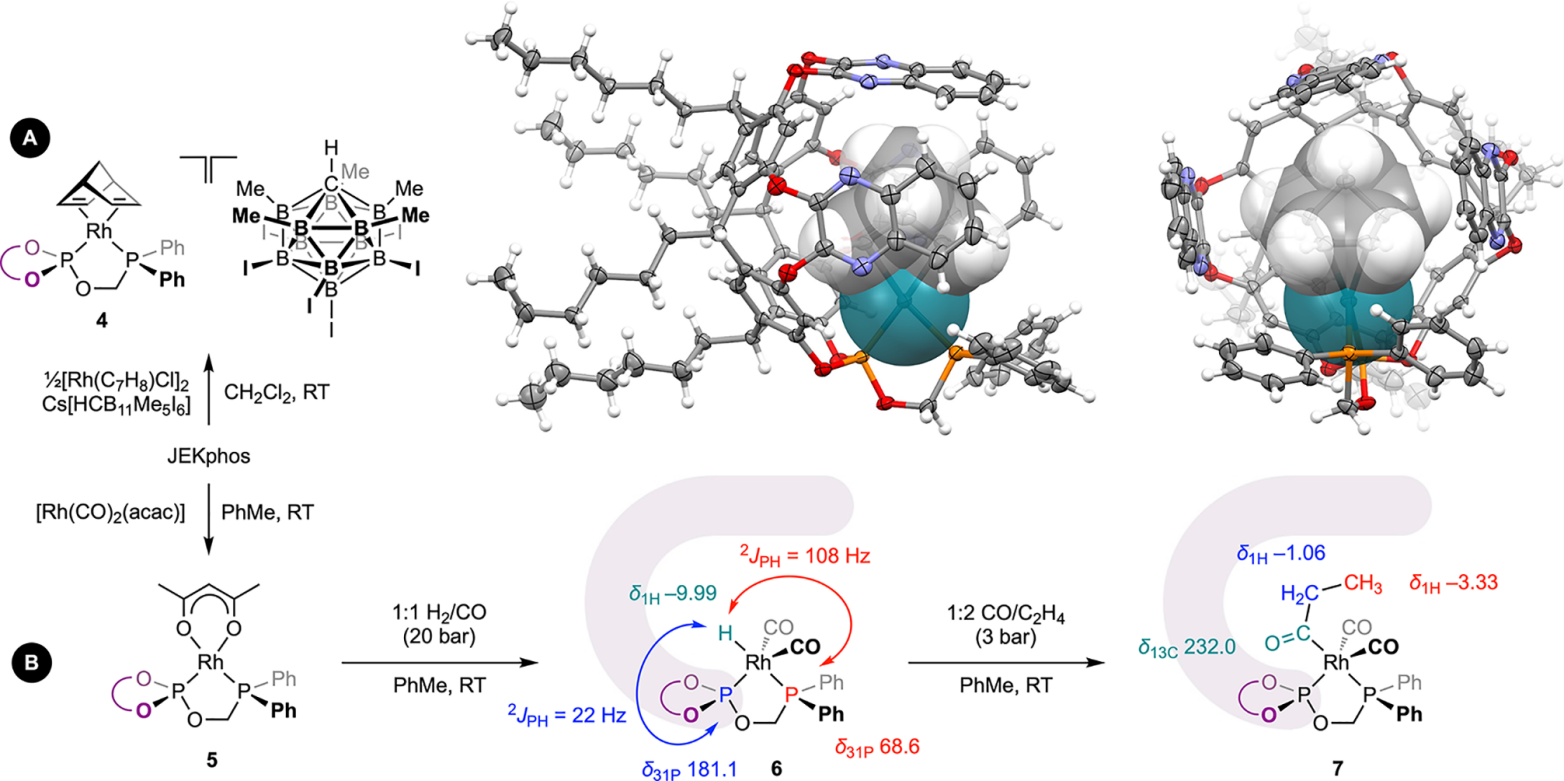
(A) Preparation and solid-state structure of model complex **4**[HCB_11_Me_5_I_6_]; thermal ellipsoids at 50% probability and the rhodium(I) norbornadiene fragment emphasised in space fill; minor disordered component (1×hexyl chain), C_6_H_5_F solvent molecules and carborane counterion omitted for clarity. Removal of the norbornadiene ligand creates a cavity of ∼106 Å^3^. (B) Preparation and reactivity of precatalyst **5**; purple shading to give impression of the resorcin[4]arene cavity.

The well-defined, neutral rhodium(I) acetyl acetonate (acac) complex [Rh(JEKphos)(acac)] **5** was chosen to explore JEKphos in hydroformylation catalysis. The analytically pure precatalyst was obtained by substitution of [Rh(acac)(CO)_2_] with the phosphite-phosphine ligand in toluene at room temperature, isolated in 80% yield, and fully characterized (δ_31P_ 160.8, ^1^*J*_RhP_ = 305 Hz; 76.6, ^1^*J*_RhP_ = 181.6 Hz; ^2^*J*_PP_ = 79 Hz; Figure 2B). Conversion of **5** into catalytically active rhodium(I) hydride complex [Rh(JEKphos)H(CO)_2_] **6** by reaction with syngas (20 bar) in toluene was verified by NMR spectroscopy within 48 h at room temperature (Figure 2B). Complex **6** is characterized by a pair of ^31^P resonances at δ 181.1 (^1^*J*_RhP_ = 225 Hz) and 68.6 (^1^*J*_RhP_ = 98 Hz; ^2^*J*_PP_ = 43 Hz), and a sharp hydride resonance at δ –9.99 that exhibits coupling to inequivalent ^31^P nuclei (^2^*J*_PH_ = 108, 22 Hz) and ^103^Rh (^1^*J*_RhH_ = 7 Hz). The trigonal bipyramidal assignment shown in Figure 2B, with the phosphine axial and the phosphite equatorial, is supported by comparison to [Rh(bobphos)H(CO)_2_] (δ_31P_ 186.9, ^1^*J*_RhP_ = 229 Hz; 108.7, ^1^*J*_RhP_ = 104 Hz; ^2^*J*_PP_ = 39 Hz; δ_1H_ –8.32, ^2^*J*_PH_ = 116, 23 Hz, ^1^*J*_RhH_ = 10 Hz)^5b^ and selective irradiation experiments, which confirm that the hydride ligand is located trans to the phosphine and, therefore, projected toward the resorcin[4]arene. Treatment of **6***in situ* with 1:2 CO/C_2_H_4_ (3 bar) resulted in conversion into the corresponding rhodium(I) acyl [Rh(JEKphos)(COEt)(CO)_2_] **7** (δ_13C_(acyl) 232.0; Figure 2B).^12^ The upfield ^1^H resonances of the methylene and methyl groups of the acyl ligand are located at δ −1.06 and −3.33, respectively, and indicate that it replaces the hydride in the axial position, deep within the cavity of the macrocycle.^11^

Building on these results, **5** was evaluated as a precatalyst for the hydroformylation of 1-hexene using a catalyst loading of 0.25 mol % and 20 bar 1:1 H_2_/CO (Table 1). Across the range 50–70 °C, **5** was found to be productive but not particularly regioselective. The *b*/*l* ratio was, however, observed to increase from 0.86 at 70 °C to 1.1 at 50 °C and appears to be inversely correlated to the turnover rate. Considerably higher branched selectivity was found using longer chain alkene substrates. Experiments conducted at 60 °C resulted in intermediate conversion and are well suited for accurate comparison of the reaction regioselectivity, with larger *b*/*l* ratios recorded in the order: 1-hexene (0.93) < 1-heptene (2.5) < 1-octene (5.9). Remarkably, for 1-octene, the branched product 2-methyloctanal was produced with 81% overall selectivity and the associated *b/l* ratio is among the highest reported for terminal alkyl alkenes.^4−6^ The increase in regioselectivity with chain length coincides with reduced turnover but, compared with the temperature dependence observed for 1-hexene, is considerably more pronounced. The catalytic activity of analogous rhodium(I) precatalysts bearing bobphos and dppe were also examined for comparison (Table 1). Under our conditions, these benchmark systems showed consistent activity and *b*/*l* ratios across the alkyl alkenes studied, with the observed regioselectivities in line with the literature.^5,13^

**Table 1:**
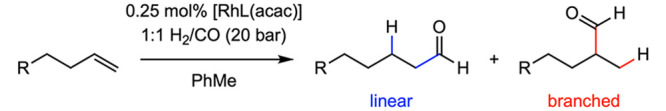
Rhodium-Catalyzed Hydroformylation of Alkyl Alkenes (Average TOF in h^–1^)^a^

		R = Et		R = Pr		R = Bu	
Ligand (L)	*T*/°C	TOF	*b*/*l*	TOF	*b*/*l*	TOF	*b*/*l*
JEKphos	50	140	1.1				
JEKphos	60	450	0.93	220	2.5	180	5.9
JEKphos	70	710	0.86				
bobphos	60	160	2.3	120	2.2	160	2.2
dppe^b^	60	71	0.41	55	0.44	73	0.42

a Conditions: 0.012 mmol of isolated precatalyst in 1.5 mL of toluene was activated at the chosen reaction temperature under 1:1 H_2_/CO (10 bar) for 30 min. The substrate was then added and the reaction ran at the chosen temperature under 1:1 H_2_/CO (20 bar) for 30 min. Conversion and product distributions were determined by ^1^H NMR analysis, are averaged over duplicate runs, and reported to 2 significant figures. Less than 10% alkene isomerization was observed in all cases.

b Reactions ran for 1.5 h to give more comparable conversions to the other systems (14–57% at 60 °C).

These results demonstrate that Rh/JEKphos is one of the few hydroformylation catalyst systems capable of producing branched aldehydes selectively from alkyl alkenes.^7^ Based on what we know about the structure/reactivity of rhodium(I) hydride complex **6** and the substrate chain length dependence, we propose that the regiocontrol is imparted by steric constraints imposed by the quinoxaline walls in the outer coordination sphere of the metal. Specifically, for the alkene substrates studied, our working hypotheses are that (a) formation of secondary alkyl intermediates by insertion of the alkene into the Rh–H bond is favored by preferential accommodation of the metal species involved in this step within the resorcin[4]arene cavity compared to those associated with the primary alkyl intermediate and (b) this effect is exacerbated as the substrate chain length increases (Figure 3). Supramolecular approaches for controlling activity and selectivity in hydroformylation reactions are attracting increasing attention,^14^ and this cavitand-based ligand design concept underscores this potential. The robust and modular nature of the JEKphos design is particularly attractive, and there is considerable scope for optimization of the catalytic performance through variation of pendant phosphine donor, the ligand backbone, and cavity size.

**Figure 3:**
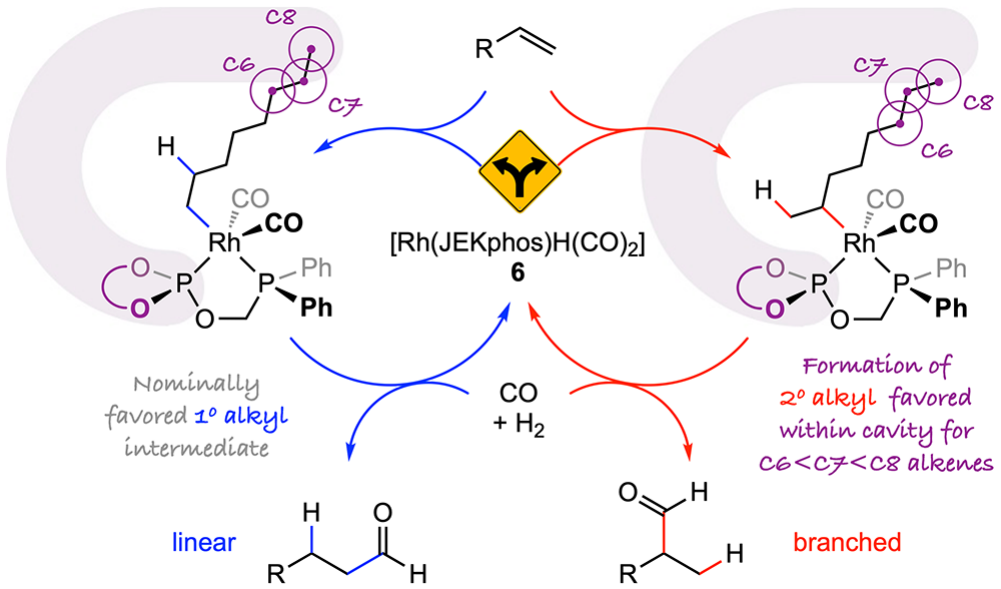
Mechanistic proposal: cavity controlled branched-selective hydroformylation of alkyl alkenes using Rh/JEKphos; purple shading to give impression of the resorcin[4]arene cavity.

## Supporting Information Available

Full experimental details and characterization data for new complexes and selected reactions, including NMR spectra of JEKphos complexes and solid-state structures of [Rh(bobphos)(acac)] and [Rh(dppe)(acac)] (PDF)

## Accession Codes

CCDC 2277328–2277330 contain the supplementary crystallographic data for this paper. These data can be obtained free of charge via www.ccdc.cam.ac.uk/data_request/cif, by emailing data_request@ccdc.cam.ac.uk, or by contacting The Cambridge Crystallographic Data Centre, 12 Union Road, Cambridge CB2 1EZ, UK; fax: +44 1223 336033.
