# Supplementary material for: A Resorcin[4]arene-Based Phosphite-Phosphine Ligand for the Branched-Selective Hydroformylation of Alkyl Alkenes
Source: ACS Catal. 2024 Jul 24;14(15):11803–7. doi: 10.1021/acscatal.4c03510 (PMC11301622; doi:10.1021/acscatal.4c03510)
Supplement: Supplementary file 1 — cs4c03510_si_001.pdf [file cs4c03510_si_001.pdf]

Supporting information for:

A Resorcin[4]arene-Based Phosphite-Phosphine Ligand for the Branched-Selective Hydroformylation of Alkyl Alkenes

Jennifer E. Smart,[a] Jack Emerson-King,[a] Rebekah J. Jeans,[b,c] Thomas M. Hood,[a] Samantha Lau,[a] Alejandro Bara-Estaún,[b] Ulrich Hintermair,[b] Paul G. Pringle,[c] and Adrian B. Chaplin[a,*]

[a] *Department of Chemistry, University of Warwick, Coventry CV4 7AL, U.K.*

E-mail: a.b.chaplin@warwick.ac.uk

[b] *Dynamic Reaction Monitoring Facility and Department of Chemistry, University of Bath, Bath BA2 7AY, U.K.*

[c] *School of Chemistry, University of Bristol, Bristol BS8 1TS, U.K.*

Table of contents

1 Synthetic experiments 2

1.1 General methods 2

1.2 Preparation of tris(quinoxaline)resorcin[4]arene phosphoramidite **2** 3

1.3 Preparation of tris(quinoxaline)resorcin[4]arene chlorophosphite **3** 4

1.4 Preparation of JEKphos 5

1.5 Preparation of [Rh(JEKphos)(nbd)][anion] **4** 7

1.6 Preparation of [Rh(JEKphos)(acac)] **5** 13

1.7 Preparation of [Rh((S_{ax},*S*,*S*)-bobphos)(acac)] 15

1.8 Preparation of [Rh(dppe)(acac)] 17

2 Hydroformylation reactions 19

2.1 General methods 19

2.2 Hydroformylation reactions 19

2.3 Reaction of [Rh(JEKphos)(acac)] **5** with syngas 28

2.4 Reaction of [Rh(JEKphos)H(CO)$_2$] **6** generated *in situ* with CO/C_2H_4 29

3 References 31

1 Synthetic experiments

1.1 General methods

All manipulations were performed under an atmosphere of argon using Schlenk and glove box techniques unless otherwise stated. Glassware was oven dried at 150 °C overnight and flame-dried under vacuum prior to use. Molecular sieves were activated by heating at 300 °C *in vacuo* overnight. C_6D_6 was dried over sodium, distilled, freeze-pump-thaw degassed and stored over a potassium mirror. CD_2Cl_2 was freeze−pump−thaw degassed and dried over 3 Å molecular sieves. C_6H_5F was pre-dried over Al_2O_3, distilled from calcium hydride, and dried over two successive batches of 3 Å molecular sieves.[1] Anhydrous toluene, THF, CH_2Cl_2, and hexane were purchased from Acros Organics or Sigma-Aldrich, freeze-pump-thaw degassed and stored over 3 Å molecular sieves. Tris(quinoxaline) substituted resorcin[4]arene **1**,[2] $[Rh(nbd)Cl]_2$ (nbd = norbornadiene),[3] $Li[Al(OR^F)_4]$ (R^F = $C(CF_3)_3$),[4] $Na[BAr^F_4]$ (Ar^F = 3,5-$(CF_3)_2C_6H_3$),[5] and $Cs[HCB_{11}Me_5I_6]$[6] were prepared using published procedures. $[Rh(CO)_2(acac)]$ (acac = acetylacetonate) and (S_{ax},*S*,*S*)-bobphos were purchased from Strem and stored under argon. All other reagents are routinely available commercial products. Triethylamine was dried over calcium hydride overnight, distilled, freeze-pump-thaw degassed and stored over 3 Å molecular sieves. $P(NMe_2)_3$ and diphenylphosphine were freeze-pump-thaw degassed and stored under argon.

NMR spectra were recorded on Bruker spectrometers (300–500 MHz) under argon at 298 K unless otherwise stated. Chemical shifts are quoted in ppm and coupling constants in Hz. ^1H and $^{13}C\{^1H\}$ NMR spectra were assigned using a combination of COSY, HSQC and HMBC correlation experiments, as well as attached proton test (APT) NMR experiments. Virtual triplets (vt) are reported as the separation between the first and third lines.[7] The quinoxaline substituents are abbreviated Qx and resorcin[4]arene aryl units Ar. Within the resolution of the experiments, it was not possible to unambiguously differentiate all of the ArO and QxO ^{13}C resonances.

Crystallographic data were collected on a Rigaku Oxford Diffraction SuperNova AtlasS2 CCD diffractometer at 150(2) K using graphite monochromated MoKα or CuKα radiation and an Oxford Cryosystems N-HeliX cryostat. Data were collected and reduced using CrysAlisPro. The structures were solved using SHELXT and refined using SHELXL, through the Olex2 interface.[8,9] Full details for all structures reported are documented in the CIF, which have been deposited with the Cambridge Crystallographic Data Centre under CCDC 2277328–2277330.

Microanalyses were performed at the London Metropolitan University by Stephen Boyer or by Elemental Microanalysis Ltd.

1.2 Preparation of tris(quinoxaline)resorcin[4]arene phosphoramidite 2

To a solution of tris(quinoxaline)resorcin[4]arene **1** (6.00 g, 5.00 mmol) in toluene (60 mL) heated at 75 °C was added Et_3N (1.74 mL, 12.5 mmol) and $P(NMe_2)_3$ (1.36 mL, 7.50 mmol). The reaction mixture was stirred for a further 1.5 h and then cooled to room temperature. Volatiles were removed *in vacuo* and the residue purified by column chromatography in air (SiO_2; 85% CH_2Cl_2,15% hexane, 1% Et_3N; R_f = 0.6) to afford the product as a white solid. Yield: 2.01 g (1.57 mmol, 32%). Spectroscopic data are in good agreement with the undecyl analogue.[10]

^{1}H NMR (400 MHz, air, $CDCl_3$): δ 8.31 (s, 2H, ArH), 8.01 (d, $^3J_{HH}$ = 8.4, 2H, QxH), 7.68–7.78 (m, 4H, 2×QxH), 7.59 (t, $^3J_{HH}$ = 7.8, 2H, QxH), 7.49 (t, $^3J_{HH}$ = 7.9, 2H, QxH), 7.36–7.43 (m, 2H, QxH), 7.23 (s, 2H, ArH), 7.21 (s, 2H, ArH), 7.20 (s, 2H, ArH), 5.69 (t, $^3J_{HH}$ = 8.2, 3H, 2×ArCHAr), 4.55 (t, $^3J_{HH}$ = 8.1, 1H, ArCHAr), 2.78 (d, $^3J_{PH}$ = 10.5, 6H, NCH_3), 2.29 (q, $^3J_{HH}$ = 7.3, 6H, 2×CHC<u>H</u>$_2$) 2.20 (q, $^3J_{HH}$ = 7.5, 2H, CHC<u>H</u>$_2$), 1.23–1.58 (m, 32H, 12×CH_2), 0.85–0.99 (m, 12H, 3×CH_3).
^{31}P{^{1}H} NMR (162 MHz, air, $CDCl_3$): δ 142.0 (s).

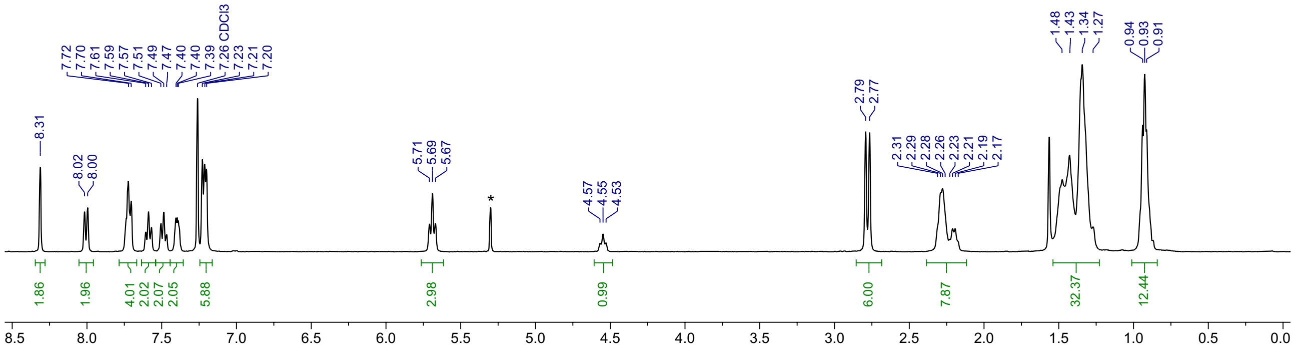

Figure S1. ^{1}H NMR spectrum of **2** (400 MHz, air, $CDCl_3$). * = residual CH_2Cl_2 solvent.

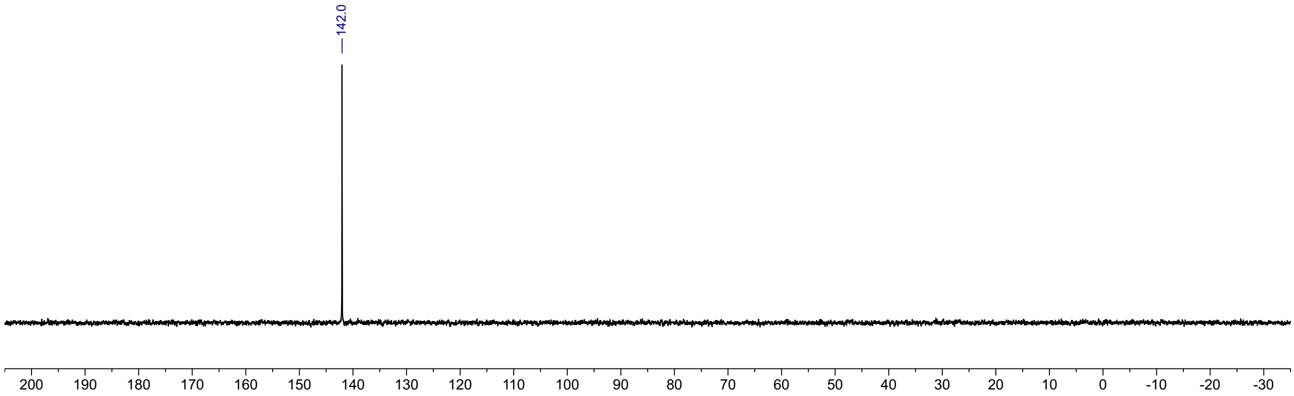

Figure S2. ^{31}P{^{1}H} NMR spectrum of **2** (162 MHz, air, $CDCl_3$).

1.3 Preparation of tris(quinoxaline)resorcin[4]arene chlorophosphite 3

To a solution of tris(quinoxaline)resorcin[4]arene phosphoramidite **2** (1.60 g, 1.25 mmol) in THF (40 mL) was added HCl (3.75 mL, 1 M in Et_2O, 3.75 mmol) and the reaction stirred for 1 h at room temperature. The resulting suspension was filtered and the product isolated as a white solid upon removal of volatiles *in vacuo*. Yield: 1.55 g (1.22 mmol, 98%).

^{1}H NMR (500 MHz, CD_2Cl_2): δ 8.24 (s, 2H, ArH), 7.93 (d, $^3J_{HH}$ = 8.4, 2H, QxH), 7.80–7.85 (m, 2H, QxH), 7.67 (d, $^3J_{HH}$ = 8.2, 2H, QxH), 7.58 (t, $^3J_{HH}$ = 7.6, 2H, QxH), 7.51–7.55 (m, 2H, QxH), 7.50 (t, $^3J_{HH}$ = 7.4, 2H, QxH), 7.29 (s, 2H, ArH), 7.25 (s, 2H, ArH), 7.22 (s, 2H, ArH), 5.72 (t, $^3J_{HH}$ = 8.1, 1H, ArCHAr), 5.66 (t, $^3J_{HH}$ = 8.2, 2H, ArCHAr), 4.49 (t, $^3J_{HH}$ = 7.9, 1H, ArCHAr), 2.20–2.38 (m, 8H, 3×CHCH$_2$), 1.30–1.55 (m, 32H, 12×CH_2), 0.89–0.97 (m, 12H, 3×CH_3).
^{13}C{^{1}H} NMR (126 MHz, CD_2Cl_2): δ 153.1 (s, CO), 153.0 (s, CO), 152.9 (s, QxO), 152.8 (s, CO), 152.7 (s, CO), 152.4 (br, ArO), 148.4 (d, $^2J_{PC}$ = 16, ArO), 140.2 (s, Qx{C}), 140.1 (s, Qx{C}), 140.0 (s, Qx{C}), 136.6 (s, ArCH), 136.5 (s, ArCH), 136.3 (br, ArCH), 135.7 (br, ArCH), 129.9 (s, QxH), 129.6 (s, QxH), 129.5 (s, QxH), 128.3 (s, QxH), 128.2 (s, QxH), 128.1 (s, QxH), 124.2 (s, ArH), 122.9 (s, ArH), 119.5 (s, ArH), 118.6 (d, $^3J_{PC}$ = 3, ArH), 37.0 (s, ArCHAr), 34.7 (s, ArCHAr), 34.6 (s, ArCHAr), 32.9 (s, CH_2), 32.7 (s, CH_2), 32.33 (s, CH_2), 32.28 (s, CH_2), 32.26 (s, CH_2), 31.3 (s, CH_2), 29.82 (s, CH_2), 29.80 (s, CH_2), 29.75 (s, CH_2), 28.44 (s, CH_2), 28.38 (s, CH_2), 28.2 (s, CH_2), 23.11 (s, CH_2), 23.09 (s, CH_2), 23.07 (s, CH_2), 14.28 (s, CH_3), 14.26 (s, CH_3), 14.25 (s, CH_3).
^{31}P{^{1}H} NMR (162 MHz, CD_2Cl_2): δ 121.3 (s).
Anal. Calcd for $C_{76}H_{76}ClN_6O_8P$ (1267.90 g·mol^{-1}): C, 72.00; H, 6.04; N, 6.63. Found: C, 71.81; H, 5.92; N, 6.47.

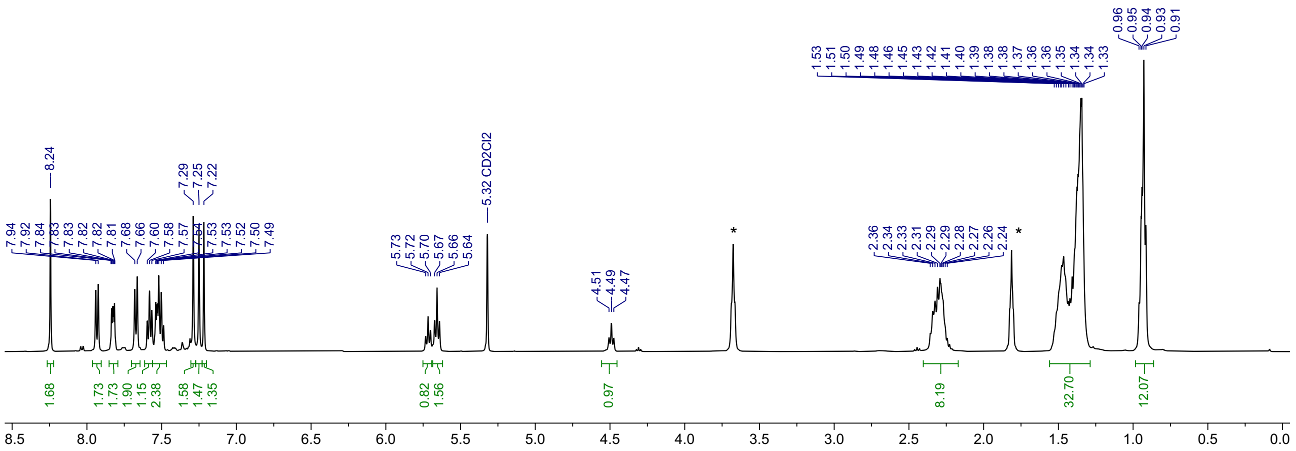

Figure S3. ^{1}H NMR spectrum of **3** (500 MHz, CD_2Cl_2). * = residual THF solvent.

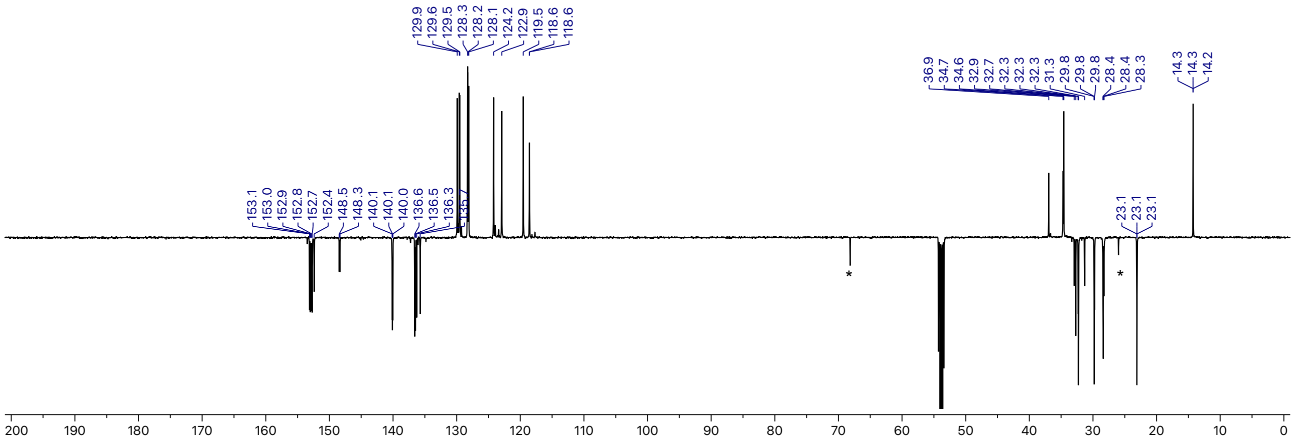

Figure S4. $^{13}C\{^{1}H\}$ APT NMR spectrum of **3** (126 MHz, CD_2Cl_2). * = residual THF solvent.

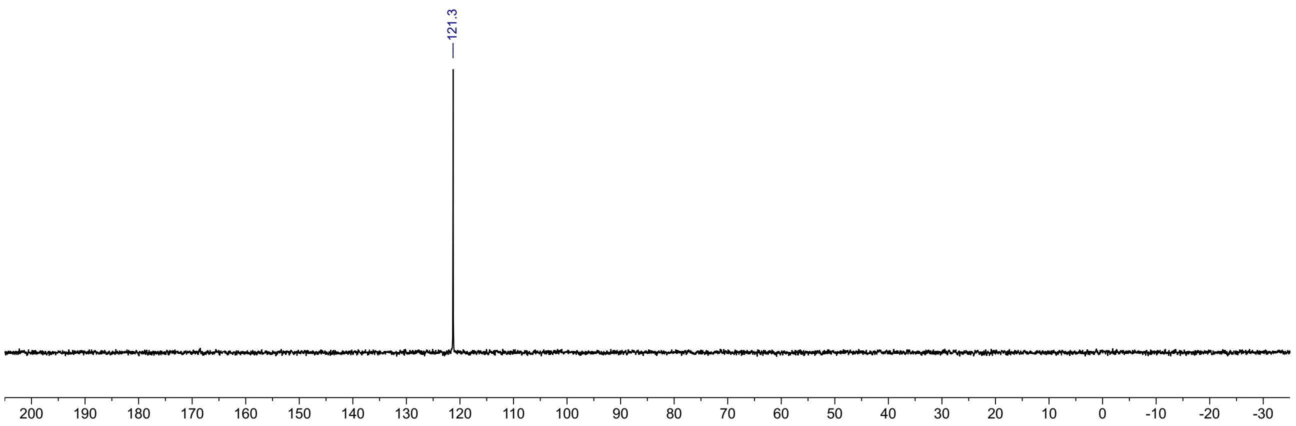

Figure S5. $^{31}P\{^{1}H\}$ NMR spectrum of **3** (162 MHz, CD_2Cl_2).

1.4 Preparation of JEKphos

A suspension of paraformaldehyde (23.7 mg, 0.790 mmol) and diphenylphosphine (137 µL, 0.79 mmol) was heated at 120 °C for 1 h. The resulting colourless oil was extracted into CH_2Cl_2 (1 mL) and added to a solution of tris(quinoxaline)resorcin[4]arene chlorophosphite **3** (1.000 g, 0.790 mmol) in CH_2Cl_2 (10 mL). Et_3N (121 µL, 0.869 mmol) was added and the reaction mixture stirred at room temperature for 1 h. Volatiles were removed *in vacuo* and the residue extracted with toluene (10 mL) to afford the product as white solid upon removal of the solvent *in vacuo* and trituration with hexane (2×10 mL). Yield: 1.005 g (0.694 mmol, 88%).

^{1}H NMR (500 MHz, CD_2Cl_2): δ 8.24 (s, 2H, ArH), 7.96 (d, $^{3}J_{HH}$ = 8.2, 2H, QxH), 7.72–7.79 (m, 4H, 2×QxH), 7.62 (t, $^{3}J_{HH}$ = 7.5, 2H, QxH), 7.54–7.59 (m, 4H, *o*-Ph), 7.53 (t, $^{3}J_{HH}$ = 7.9, 2H, QxH), 7.47–7.51 (m, 2H, QxH), 7.40–7.45 (m, 6H, *m*-Ph+*p*-Ph), 7.31 (s, 2H, ArH), 7.29 (s, 2H, ArH), 7.22 (s, 2H, ArH), 5.68 (t, $^{3}J_{HH}$ = 8.2, 3H, 2×ArCHAr), 5.03 (app t, J = 5.9, 2H, PCH_2), 4.51 (t, $^{3}J_{HH}$ = 7.9, 1H, ArCHAr), 2.27–2.38 (m, 6H, 2×CHC$\underline{H}_2$), 2.23 (q, $^{3}J_{HH}$ = 8.0, 2H, CHC$\underline{H}_2$), 1.25–1.55 (m, 32H, 12×CH_2), 0.87–0.98 (m, 12H, 3×CH_3).

$^{13}C\{^1H\}$ NMR (126 MHz, CD_2Cl_2): δ 153.1 (s, CO), 152.9 (s, CO), 152.82 (s, CO), 152.79 (s, CO), 152.76 (s, CO), 152.7 (br, ArO), 147.2 (d, $^2J_{PC}$ = 5, ArO), 140.09 (s, Qx{C}), 140.07 (s, Qx{C}), 140.05 (s, Qx{C}), 137.8 (d, $^3J_{PC}$ = 3, <u>Ar</u>CH), 136.7 (s, <u>Ar</u>CH), 136.5 (s, <u>Ar</u>CH), 135.8 (d, $^1J_{PC}$ = 11, *i*-Ph), 135.6 (br, <u>Ar</u>CH), 133.7 (d, $^2J_{PC}$ = 19, *o*-Ph), 129.8 (s, QxH), 129.7 (s, QxH), 129.53 (s, *p*-Ph), 129.48 (s, QxH), 129.0 (d, $^3J_{PC}$ = 7, *m*-Ph), 128.3 (s, QxH), 128.2 (s, QxH), 128.1 (s, QxH), 123.9 (s, ArH), 123.1 (s, ArH), 119.4 (s, ArH), 117.9 (d, $^3J_{PC}$ = 2, ArH), 62.8 (dd, $^1J_{PC}$ = 15, $^2J_{PC}$ = 5, PCH_2), 36.3 (s, Ar<u>C</u>HAr), 34.8 (s, Ar<u>C</u>HAr), 34.6 (s, Ar<u>C</u>HAr), 33.1 (s, CH_2), 32.5 (s, CH_2), 32.32 (s, CH_2), 32.30 (s, CH_2), 32.2 (s, CH_2), 31.9 (s, CH_2), 29.8 (s, 2×CH_2), 29.7 (s, CH_2), 28.44 (s, CH_2), 28.41 (s, CH_2), 28.2 (s, CH_2), 23.11 (s, CH_2), 23.10 (s, CH_2), 23.09 (s, CH_2), 14.28 (s, CH_3), 14.27 (s, CH_3), 14.25 (s, CH_3).

$^{31}P\{^1H\}$ NMR (162 MHz, CD_2Cl_2): δ 127.5 (d, $^3J_{PP}$ = 5, 1P, phosphite), −14.5 (d, $^3J_{PP}$ = 5, 1P, phosphine).

Anal. Calcd for $C_{89}H_{88}N_6O_9P_2$ (1447.66 g·mol^{-1}): C, 73.84; H, 6.13; N, 5.81. Found: C, 73.66; H, 6.16; N, 5.71.

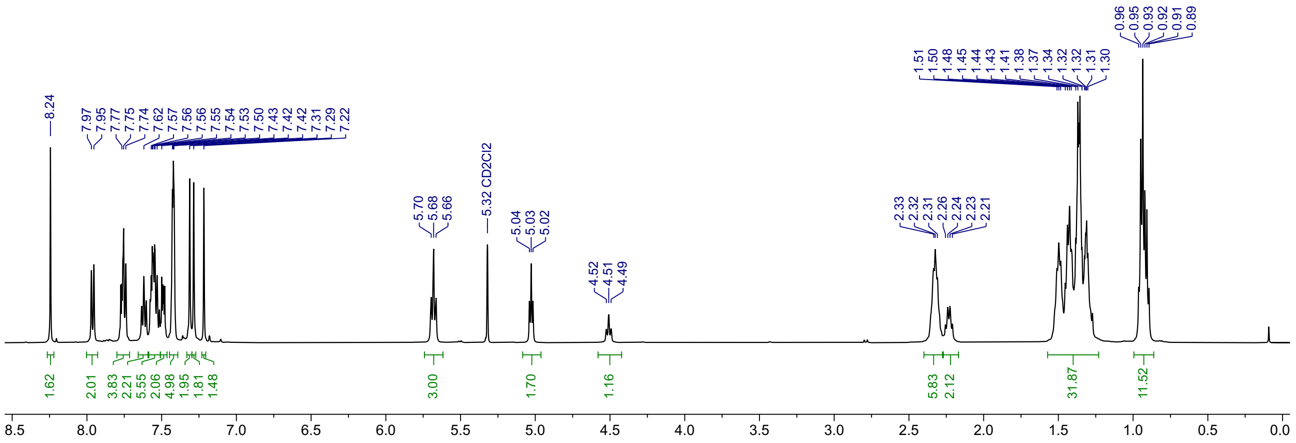

Figure S6. ^1H NMR spectrum of JEKphos (500 MHz, CD_2Cl_2).

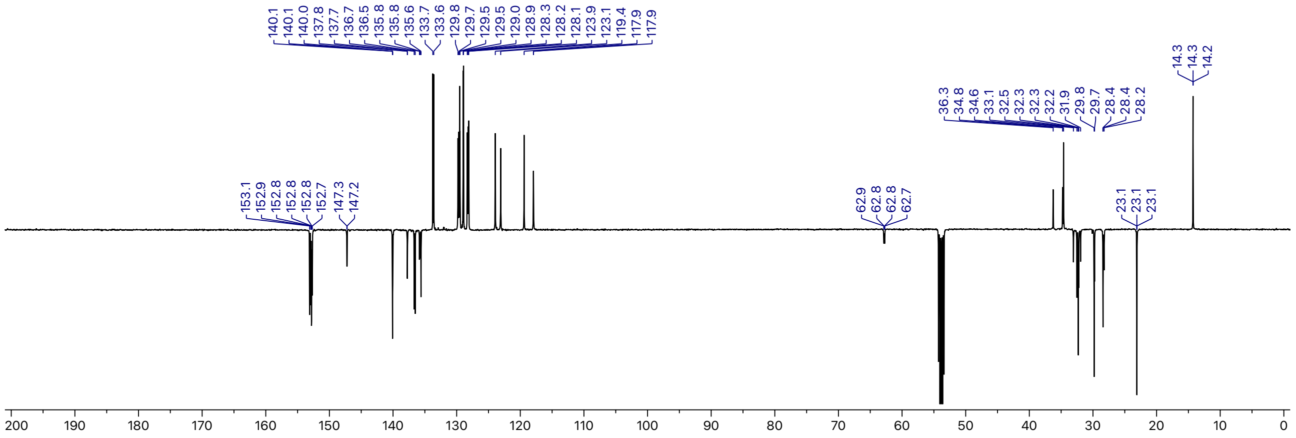

Figure S7. $^{13}C\{^1H\}$ APT NMR spectrum of JEKphos (126 MHz, CD_2Cl_2).

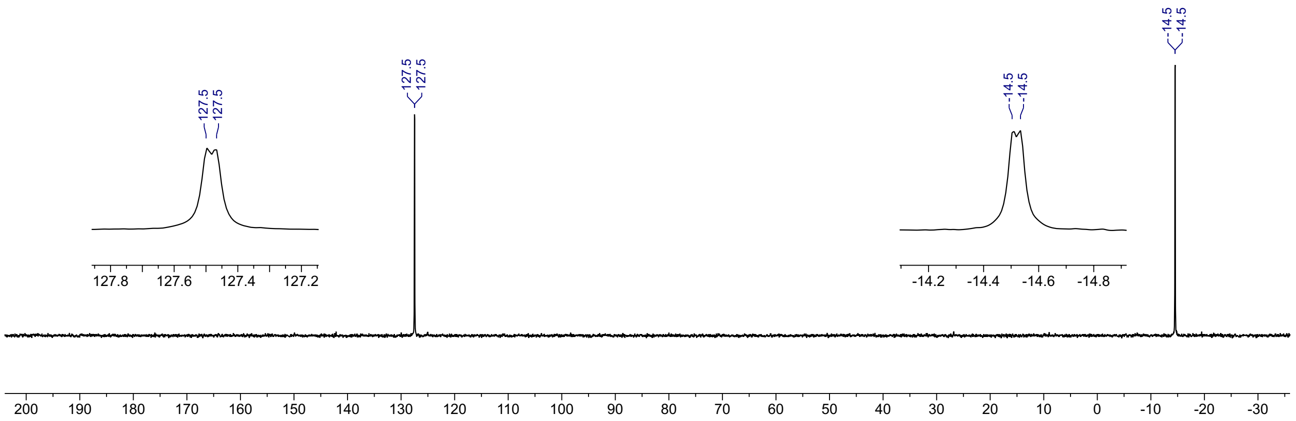

Figure S8. $^{31}P\{^1H\}$ NMR spectrum of JEKphos (162 MHz, CD_2Cl_2).

1.5 Preparation of [Rh(JEKphos)(nbd)][anion] 4

General procedure: A suspension of $[Rh(nbd)Cl]_2$, JEKphos, and M[anion] in CH_2Cl_2 was stirred at room temperature for 18 h. Excess hexane (*ca*. 10× reaction volume) was added and resulting precipitate collected by filtration. The product was isolated an orange solid by extraction into a minimal amount of CH_2Cl_2 and removal of the solvent *in vacuo*.

$[Rh(JEKphos)(nbd)][Al(OR^F)_4]$. Prepared following the general procedure using $[Rh(nbd)Cl]_2$ (23.0 mg, 50.0 µmol), JEKphos (144.8 mg, 100.0 µmol), and $Li[Al(OR^F)_4]$ (97.4 mg, 110 µmol) in CH_2Cl_2 (5 mL). Yield: 227.4 mg (87.1 µmol, 87%).

^{1}H NMR (500 MHz, CD_2Cl_2): δ 8.21 (s, 2H, ArH), 8.06 (d, $^3J_{HH}$ = 8.3, 2H, QxH), 7.78–7.86 (m, 4H, *p*-Ph+QxH), 7.77 (td, $^3J_{HH}$ = 7.3, $^4J_{PH}$ = 4.1, 4H, *m*-Ph), 7.67 (t, $^3J_{HH}$ = 7.6, 2H, QxH), 7.55 (s, 1H, ArH), 7.50–7.54 (m, 2H, QxH), 7.48 (d, $^3J_{HH}$ = 7.6, 2H, QxH), 7.47 (s, 2H, ArH), 7.42 (t, $^3J_{HH}$ = 7.6, 2H, QxH), 7.35–7.41 (m, 4H, *o*-Ph), 7.39 (s, ArH), 5.78 (t, $^3J_{HH}$ = 8.1, 2H, ArCHAr), 5.62 (t, $^3J_{HH}$ = 8.1, 1H, ArCHAr), 4.58 (d, $^2J_{PH}$ = 23.5, 2H, PCH_2), 4.46 (t, $^3J_{HH}$ = 7.4, 1H, ArCHAr), 3.64 (br, 2H, nbd{CH=CH}), 2.35–2.45 (m, 4H, CHC$\underline{H}_2$), 2.34 (q, $^3J_{HH}$ = 7.3, 2H, CHC$\underline{H}_2$), 2.28 (q, $^3J_{HH}$ = 7.4, 2H, CHC$\underline{H}_2$), 1.91 (br, 2H, nbd{CH=CH}), 1.20–1.58 (m, 32H, 12×CH_2), 0.86–0.97 (m, 12H, 3×CH_3), –0.31 (s, 2H, nbd{CH}), –2.22 (br d, $^2J_{HH}$ ~ 7, 1H, nbd{CH_2}), –2.67 (br d, $^2J_{HH}$ ~ 7, 1H, nbd{CH_2}).
^{13}C{^{1}H} NMR (126 MHz, CD_2Cl_2): δ 153.22 (s, CO), 153.20 (s, CO), 153.15 (s, CO), 153.1 (s, CO), 152.7 (s, ArO), 152.6 (s, ArO), 146.2 (s, ArO), 140.3 (s, Qx{C}), 140.1 (s, Qx{C}), 140.0 (s, Qx{C}), 139.1 (br, $\underline{Ar}$CH), 137.1, (s, $\underline{Ar}$CH), 136.6, (s, $\underline{Ar}$CH), 136.2 (d, $^3J_{PC}$ = 3, $\underline{Ar}$CH), 133.8 (d, $^4J_{PC}$ = 2, *p*-Ph), 132.8 (d, $^2J_{PC}$ = 11, *o*-Ph), 130.9 (d, $^3J_{PC}$ = 11, *m*-Ph), 130.7 (s, QxH), 130.6 (s, QxH), 130.1 (s, QxH), 128.6 (s, QxH), 127.9 (s, QxH), 127.8 (s, QxH), 126.1 (d, $^1J_{PC}$ = 47, *i*-Ph), 124.8 (s, ArH), 123.8 (s, ArH), 121.7 (q, $^1J_{FC}$ = 292, CF_3), 118.7 (s, ArH), 117.8 (d, $^3J_{PC}$ = 4, ArH), 96.9 (br, nbd{CH=CH}), 96.8 (br, nbd{CH=CH}), 69.8 (br, nbd{CH_2}), 68.2 (dd, $^2J_{PC}$ = 30, $^2J_{PC}$ = 16, PCH_2),53.6 (obs, nbd{CH}),36.5 (s, Ar$\underline{C}$HAr), 34.89 (s, Ar$\underline{C}$HAr), 34.87 (s, Ar$\underline{C}$HAr), 32.8 (s, CH_2), 32.33 (s, CH_2),32.26 (s, CH_2), 32.25 (s, CH_2), 32.17 (s, CH_2), 32.15 (s, CH_2), 29.72 (s, CH_2), 29.70 (s, CH_2),

29.6 (s, CH_2), 28.3 (s, 2×CH_2), 28.1 (s, CH_2), 23.08 (s, CH_2), 23.07 (s, CH_2), 23.0 (s, CH_2), 14.25 (s, CH_3), 14.24 (s, CH_3), 14.19 (s, CH_3). The <u>C</u>(CF_3)$_3$ signal was not located.

^{31}P{^{1}H} NMR (162 MHz, CD_2Cl_2): δ 160.8 (dd, $^1J_{RhP}$ = 263, $^2J_{PP}$ = 51, 1P, phosphite), 65.7 (dd, $^1J_{RhP}$ = 154, $^2J_{PP}$ = 51, 1P, phosphine).

^{19}F{^{1}H} NMR (282 MHz, CD_2Cl_2): δ –75.69 (s)

Anal. Calcd for $C_{112}H_{96}AlF_{36}N_6O_{13}P_2Rh$ (2609.81 g·mol^{-1}): C, 51.55; H, 3.71; N, 3.22. Found: C, 51.45; H, 3.63; N, 3.19.

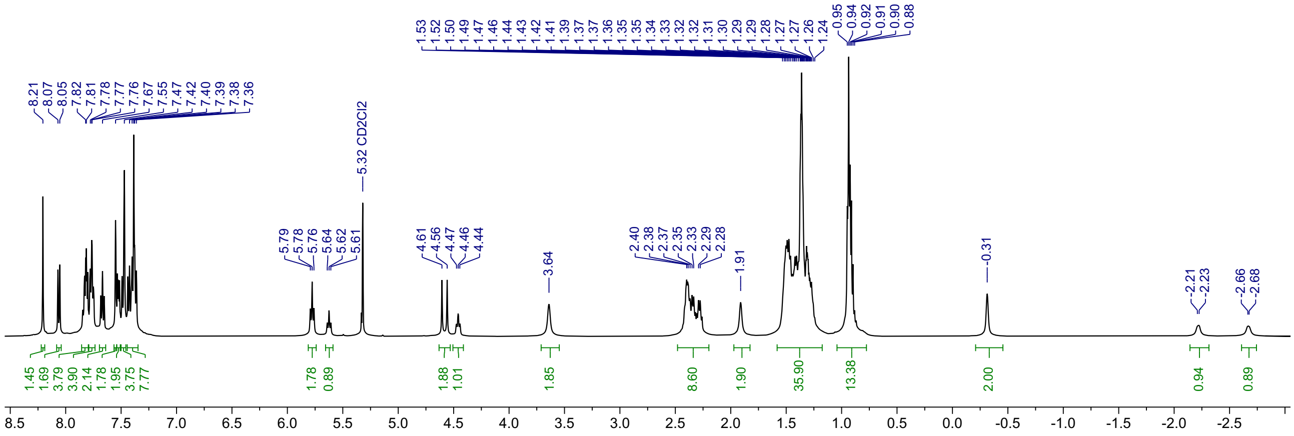

Figure S9. ^{1}H NMR spectrum of [Rh(JEKphos)(nbd)][Al(OR^F)$_4$] (500 MHz, CD_2Cl_2).

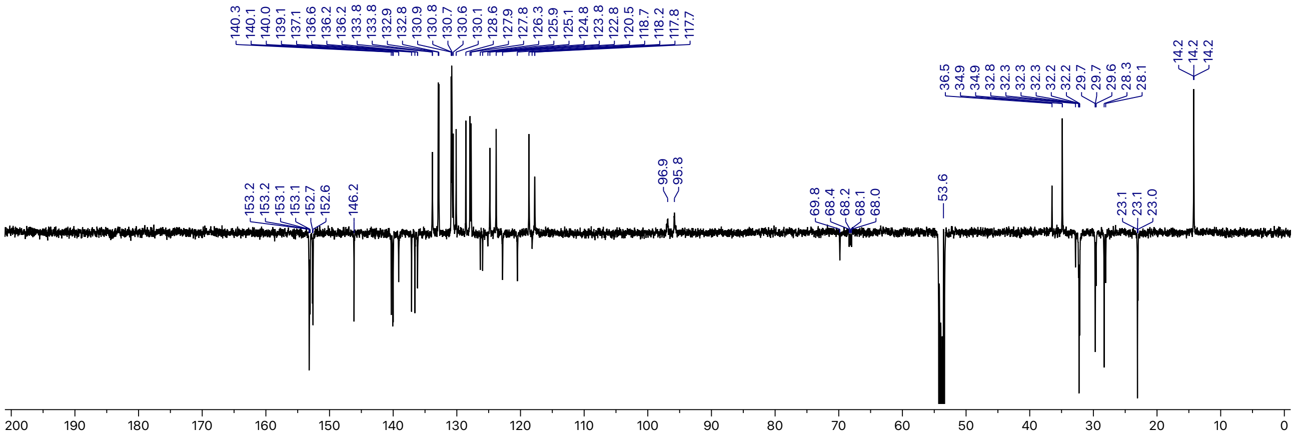

Figure S10. ^{13}C{^{1}H} APT NMR spectrum of [Rh(JEKphos)(nbd)][Al(OR^F)$_4$] (126 MHz, CD_2Cl_2).

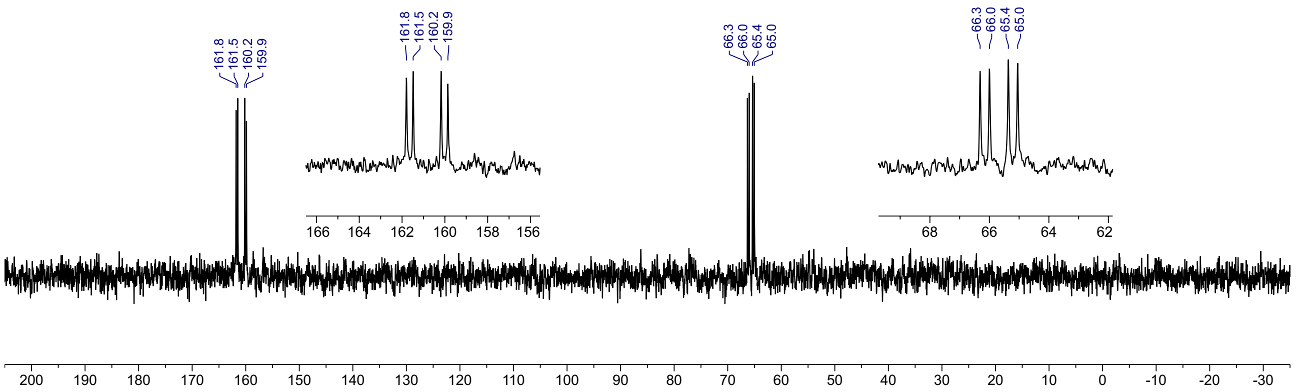

Figure S11. $^{31}P\{^1H\}$ NMR spectrum of [Rh(JEKphos)(nbd)][Al(OR^F)$_4$] (162 MHz, CD_2Cl_2).

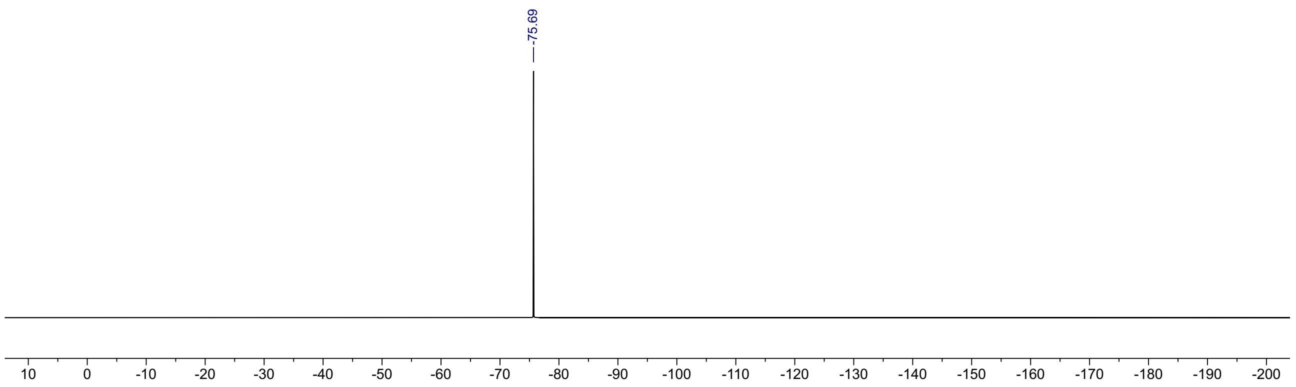

Figure S12. $^{19}F\{^1H\}$ NMR spectrum of [Rh(JEKphos)(nbd)][Al(OR^F)$_4$] (282 MHz, CD_2Cl_2).

[Rh(JEKphos)(nbd)][BAr$^F{}_4$]. Prepared following the general procedure using $[Rh(nbd)Cl]_2$ (11.5 mg, 25.0 μmol), JEKphos (77.4 mg, 50.0 μmol), and Na[BAr$^F{}_4$] (21.2 mg, 50.0 μmol) in CH_2Cl_2 (5 mL). Yield: 42.5 mg (17.0 μmol, 34%).

^{1}H NMR (500 MHz, CD_2Cl_2): δ 8.20 (s, 2H, ArH), 8.06 (d, $^3J_{HH}$ = 8.3, 2H, QxH), 7.77–7.83 (m, 4H, *p*-Ph+QxH), 7.70–7.77 (m, 12H, *m*-Ph+Ar^F), 7.65 (t, $^3J_{HH}$ = 7.7, 2H, QxH), 7.56 (br, 6H, ArH+Ar^F), 7.47–7.53 (m, 4H, 2×QxH), 7.47 (s, 2H, ArH), 7.39 (s, ArH), 7.35–7.43 (m, 6H, *o*-Ph+QxH), 5.77 (t, $^3J_{HH}$ = 8.1, 2H, ArCHAr), 5.61 (t, $^3J_{HH}$ = 8.1, 1H, ArCHAr), 4.58 (d, $^2J_{PH}$ = 23.7, 2H, PCH_2), 4.46 (t, $^3J_{HH}$ = 7.3, 1H, ArCHAr), 3.63 (br, 2H, nbd{CH=CH}), 2.35–2.45 (m, 4H, $CHC\underline{H}_2$), 2.34 (q, $^3J_{HH}$ = 7.3, 2H, $CHC\underline{H}_2$), 2.28 (q, $^3J_{HH}$ = 7.5, 2H, $CHC\underline{H}_2$), 1.90 (br, 2H, nbd{CH=CH}), 1.21–1.57 (m, 32H, 12×CH_2), 0.86–0.98 (m, 12H, 3×CH_3), –0.30 (s, 2H, nbd{CH}), –2.20 (br d, $^2J_{HH}$ ~ 7, 1H, nbd{CH_2}), –2.64 (br d, $^2J_{HH}$ ~ 7, 1H, nbd{CH_2}).

^{31}P{^{1}H} NMR (162 MHz, CD_2Cl_2): δ 160.9 (dd, $^1J_{RhP}$ = 263, $^2J_{PP}$ = 51, 1P, phosphite), 65.7 (dd, $^1J_{RhP}$ = 153, $^2J_{PP}$ = 51, 1P, phosphine).

^{19}F{^{1}H} NMR (282 MHz, CD_2Cl_2): δ –62.86 (s).

^{11}B{^{1}H} NMR (96 MHz, CD_2Cl_2): δ –6.6 (s).

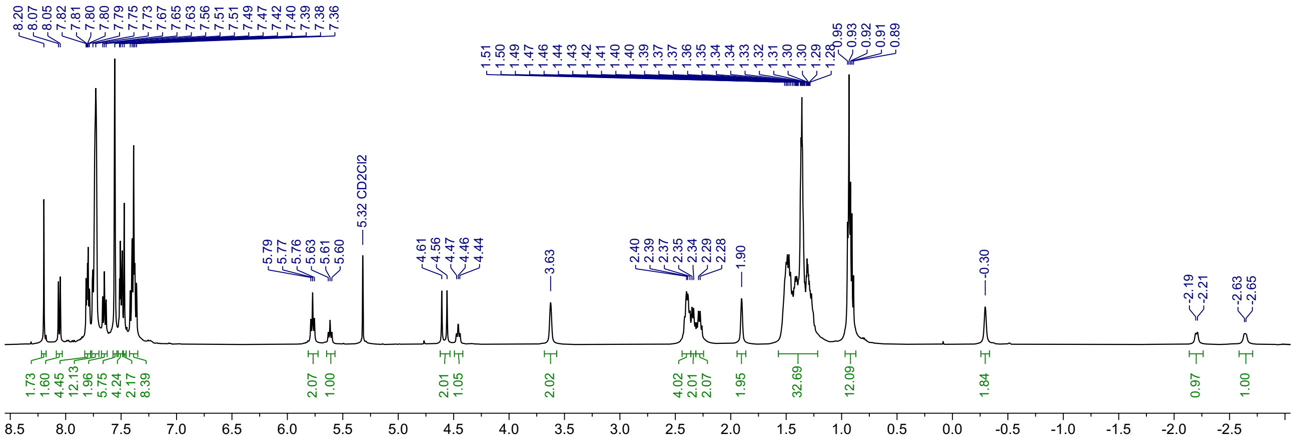

Figure S13. ^{1}H NMR spectrum of [Rh(JEKphos)(nbd)][BAr$^{F}_{4}$] (500 MHz, CD_2Cl_2).

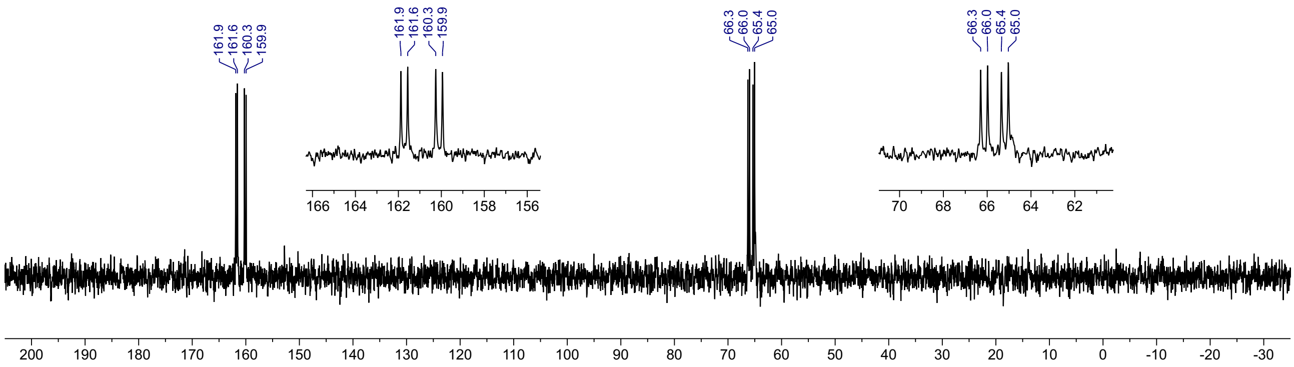

Figure S14. $^{31}P\{^{1}H\}$ NMR spectrum of [Rh(JEKphos)(nbd)][BAr$^{F}_{4}$] (162 MHz, CD_2Cl_2).

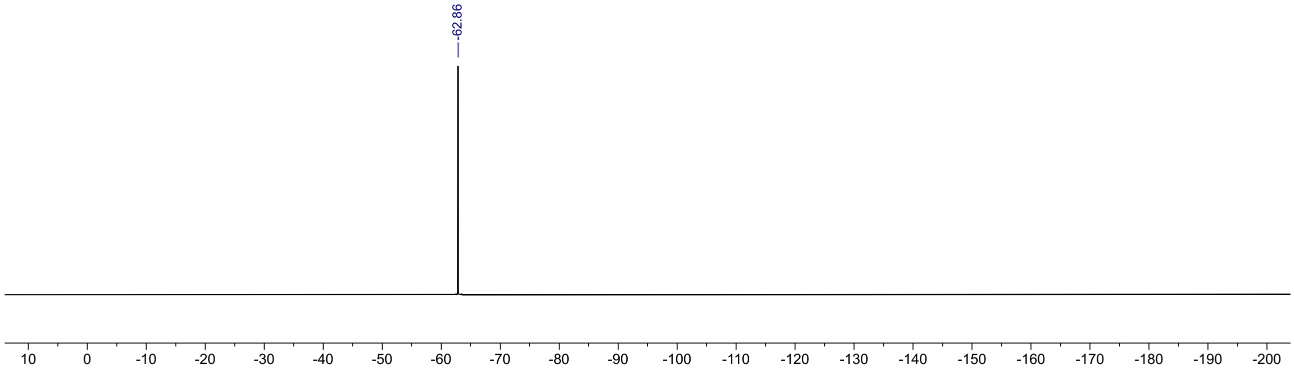

Figure S15. $^{19}F\{^{1}H\}$ NMR spectrum of [Rh(JEKphos)(nbd)][BAr$^{F}_{4}$] (282 MHz, CD_2Cl_2).

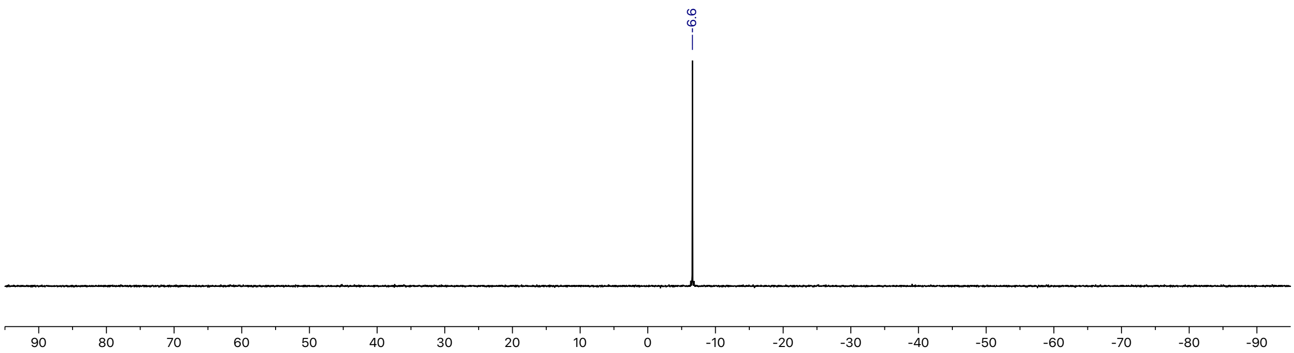

Figure S16. $^{11}B\{^1H\}$ NMR spectrum of [Rh(JEKphos)(nbd)][BAr$^F{}_4$] (96 MHz, CD_2Cl_2).

[Rh(JEKphos)(nbd)][$HCB_{11}Me_5I_6$]. Prepared following the general procedure using [Rh(nbd)Cl]$_2$ (23.0 mg, 50.0 µmol), JEKphos (144.8 mg, 100.0 µmol), and Cs[$HCB_{11}Me_5I_6$] (121.2 mg, 110.0 µmol) in CH_2Cl_2 (5 mL). Yield: 121.9 mg (46.7 µmol, 47%). Single crystals suitable for X-ray diffraction were obtained by slow diffusion of hexane into a C_6H_5F solution at room temperature.

^{1}H NMR (500 MHz, CD_2Cl_2): δ 8.21 (s, 2H, ArH), 8.06 (d, $^3J_{HH}$ = 8.3, 2H, QxH), 7.85 (t, $^3J_{HH}$ = 7.6, 2H, *p*-Ph), 7.77–7.83 (m, 6H, *m*-Ph+QxH), 7.74 (t, $^3J_{HH}$ = 7.6, 2H, QxH), 7.59–7.63 (m, 2H, QxH), 7.55 (s, 1H, ArH), 7.50 (t, $^3J_{HH}$ = 7.3, 2H, QxH), 7.46 (s, 2H, ArH), 7.46 (d, $^3J_{HH}$ = 7.4, 2H, QxH),7.41 (dd, $^3J_{PH}$ = 11.6, $^3J_{HH}$ = 7.4, 4H, *o*-Ph), 7.38 (s, ArH), 5.77 (t, $^3J_{HH}$ = 8.1, 2H, ArCHAr), 5.63 (t, $^3J_{HH}$ = 8.2, 1H, ArCHAr), 4.61 (d, $^2J_{PH}$ = 23.4, 2H, PCH_2), 4.46 (t, $^3J_{HH}$ = 7.4, 1H, ArCHAr), 3.69 (br, 2H, nbd{CH=CH}), 2.71 (s, 1H, $HCB_{11}Me_5I$), (2.35–2.46 (m, 4H, CHC$\underline{H}_2$), 2.34 (q, $^3J_{HH}$ = 7.2, 2H, CHC$\underline{H}_2$), 2.28 (q, $^3J_{HH}$ = 7.4, 2H, CHC$\underline{H}_2$), 1.92 (br, 2H, nbd{CH=CH}), 1.15–1.57 (m, 32H, 12×CH_2), 0.85–0.97 (m, 12H, 3×CH_3), 0.29 (s, 15H, BMe), –0.32 (s, 2H, nbd{CH}), –2.21 (vbr, fwhm = 34 Hz,1H, nbd{CH_2}), –2.69 (vbr, fwhm = 36 Hz, 1H, nbd{CH_2}).

^{31}P{^{1}H} NMR (162 MHz, CD_2Cl_2): δ 160.8 (dd, $^1J_{RhP}$ = 263, $^2J_{PP}$ = 51, 1P, phosphite), 65.7 (dd, $^1J_{RhP}$ = 153, $^2J_{PP}$ = 51, 1P, phosphine).

^{11}B{^{1}H} NMR (96 MHz, CD_2Cl_2): δ –8.8 (br, fwhm = 166 Hz, 6B, BI), –16.4 (br, fwhm = 79 Hz, 5B, BMe).

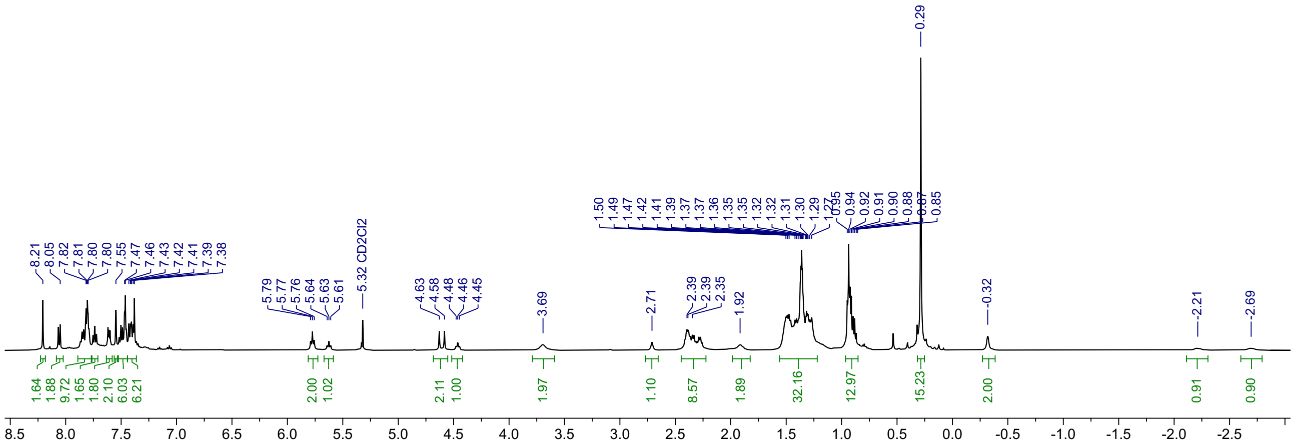

Figure S17. ^{1}H NMR spectrum of [Rh(JEKphos)(nbd)][$HCB_{11}Me_5I_6$] (500 MHz, CD_2Cl_2).

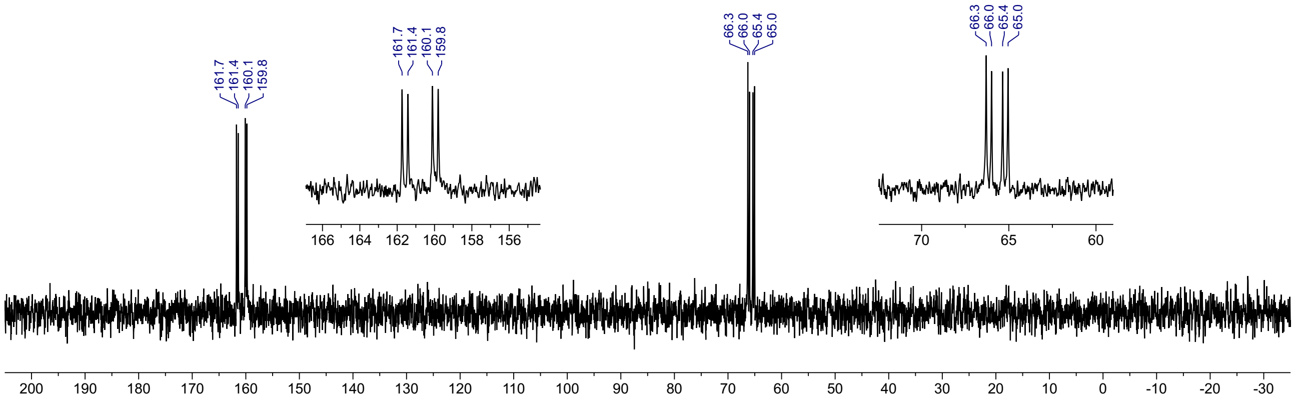

Figure S18. ^{31}P{^{1}H} NMR spectrum of [Rh(JEKphos)(nbd)][$HCB_{11}Me_5I_6$] (162 MHz, CD_2Cl_2).

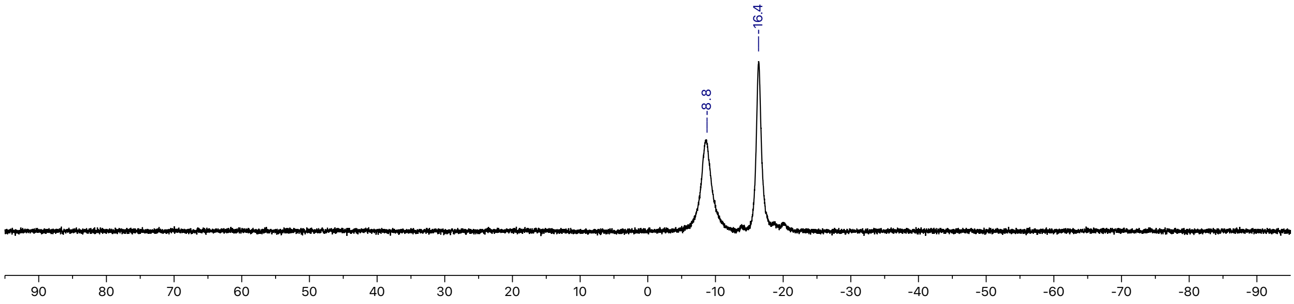

Figure S19. ^{11}B{^{1}H} NMR spectrum of [Rh(JEKphos)(nbd)][$HCB_{11}Me_5I_6$] (96 MHz, CD_2Cl_2).

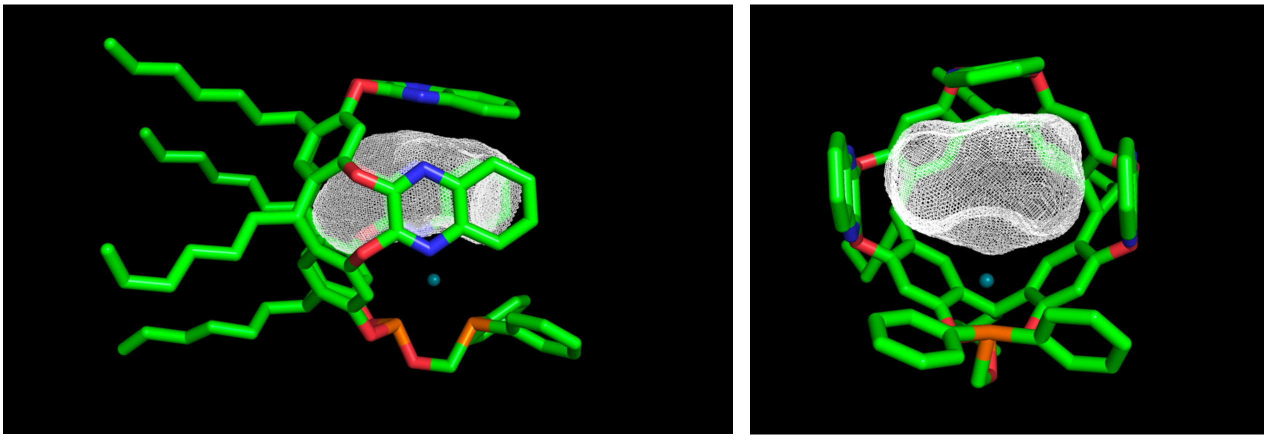

Figure S20. MoloVol 1.1.0 analysis of the cavity created by deletion of the nbd ligand from the solid-state structure of $[Rh(JEKphos)(nbd)]^+$. Small Probe radius = 1.2 Å, Large Probe radius = 2 Å, Grid resolution = 0.1 Å, Optimisation depth = 4; Occupied volume = 105.728 $Å^3$.[11]

1.6 Preparation of [Rh(JEKphos)(acac)] 5

A solution of JEKphos (0.100 g, 69.1 µmol) in toluene (10 mL) was added to a flask charged with $[Rh(CO)_2(acac)]$ (0.0178 g, 69.0 µmol) and the resulting green solution stirred at room temperature for 2.5 h. Volatiles were removed *in vacuo* to give a green residue, which was washed with hexane (2×5 mL) and -78 °C and dried to afford the product as a pale yellow solid. Yield: 0.0918 g (55.6 µmol, 80%).

^{1}H NMR (500 MHz, C_6D_6): δ 8.95 (s, 2H, ArH), 8.15 (s, 2H, ArH), 7.96–8.00 (m, 2H, QxH), 7.83–7.91 (m, 6H, *o*-Ph+QxH), 7.56 (s, 2H, ArH), 7.54 (s, 2H, ArH), 7.46 (d, $^3J_{HH}$ = 8.1, 2H, QxH), 7.25 (t, $^3J_{HH}$ = 7.3, 4H, *m*-Ph), 7.17 (obs, *p*-Ph), 7.10–7.16 (obs m, 2H, QxH), 7.07 (app t, $^3J_{HH}$ = 7.3, 2H, QxH), 7.01 (app t, $^3J_{HH}$ = 7.2, 2H, QxH), 6.06 (t, $^3J_{HH}$ = 8.3, 1H, ArCHAr), 6.05 (t, $^3J_{HH}$ = 8.3, 2H, ArCHAr), 5.27 (t, $^3J_{HH}$ = 7.9, 1H, ArCHAr), 4.19 (d, $^2J_{PH}$ = 19.8, 2H, PCH_2), 2.32 (q, $^3J_{HH}$ = 8.1, 8H, 3×$CHC\underline{H}_2$), 1.16–1.49 (m, 32H, 12×CH_2), 0.95 (s, 1H, C(O)CH), 0.86–0.94 (m, 12H, 3×CH_3), 0.00 (s, 3H, C(O)CH_3), –2.74 (s, 3H, C(O)CH_3).

^{13}C{^{1}H} NMR (126 MHz, C_6D_6): δ 182.3 (s, C=O), 181.6 (s, C=O), 153.8 (s, CO), 153.57 (s, CO), 153.54 (s, CO), 153.2 (s, CO), 152.8 (s, CO), 152.5 (br, ArO), 148.1 (s, ArO), 140.4 (s, Qx{C}), 140.2 (s, Qx{C}), 140.0 (s, Qx{C}), 137.11 (br, $\underline{Ar}$CH), 137.09 (s, $\underline{Ar}$CH), 136.4 (s, $\underline{Ar}$CH), 134.9 (s, $\underline{Ar}$CH), 134.7 (d, $^1J_{PC}$ = 43, *i*-Ph), 133.6 (d, $^2J_{PC}$ = 11, *o*-Ph), 130.4 (br, *p*-Ph), 128.9 (s, QxH), 128.68 (s, QxH), 128.66 (s, QxH), 128.63 (s, QxH), 128.5 (d, $^3J_{PC}$ = 10, *m*-Ph), 124.3 (s, ArH), 122.1 (s, ArH), 120.5 (br, ArH), 120.4 (s, ArH), 94.1 (d, $^3J_{RhC}$ = 2, C(O)$\underline{C}$H), 69.3 (dd, $^1J_{PC}$ = 34, $^2J_{PC}$ = 18, PCH_2), 36.5 (s, Ar$\underline{C}$HAr), 34.85 (s, Ar$\underline{C}$HAr), 34.76 (s, Ar$\underline{C}$HAr), 33.2 (s, CH_2), 32.6 (s, CH_2), 32.30 (s, CH_2), 32.27 (s, CH_2), 32.23 (s, CH_2), 31.5 (s, CH_2), 29.87 (s, CH_2), 29.84 (s, CH_2), 29.72 (s, CH_2), 28.43 (s, CH_2), 28.41 (s, CH_2), 28.3 (s, CH_2), 26.8 (d, $^4J_{PC}$ = 7, C(O)$\underline{C}H_3$), 23.08 (s, CH_2), 23.07 (s, CH_2), 23.02 (s, CH_2), 21.4 (d, $^4J_{PC}$ = 5, C(O)$\underline{C}H_3$), 14.33 (s, 1/2×CH_3), 14.31 (s, 1/2×CH_3). Not all QxH signals were unambiguously located.

$^{31}P\{^1H\}$ NMR (162 MHz, C_6D_6): δ 160.8 (dd, $^1J_{RhP}$ = 305, $^2J_{PP}$ = 79, 1P, phosphite), 76.6 (dd, $^1J_{RhP}$ = 181.6, $^2J_{PP}$ = 79, 1P, phosphine).

Anal. Calcd for $C_{94}H_{95}N_6O_{11}P_2Rh$ (1649.68 g·mol^{-1}): C, 68.44; H, 5.80; N, 5.09. Found: C, 68.06; H, 5.80; N, 5.00.

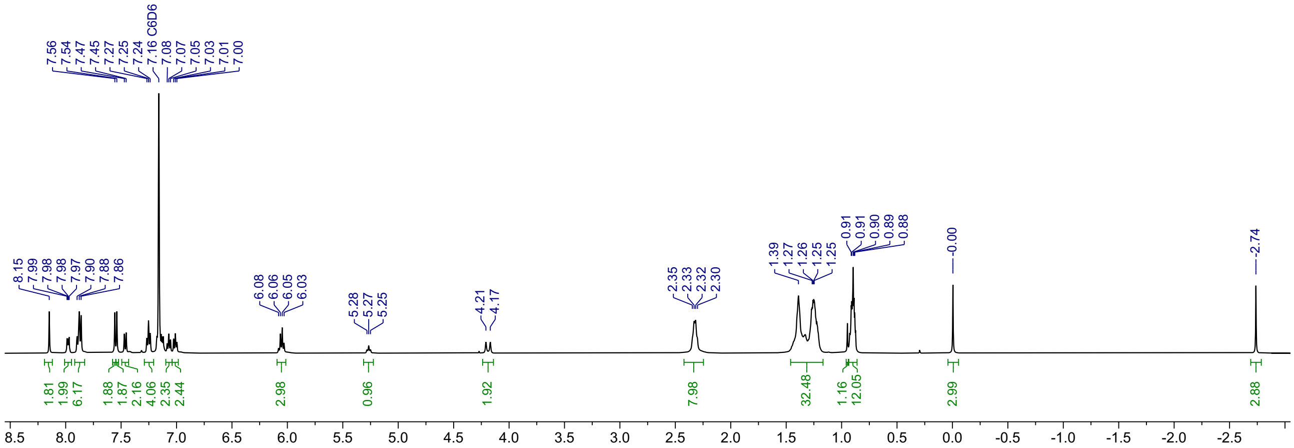

Figure S21. ^1H NMR spectrum of [Rh(JEKphos)(acac)] (500 MHz, C_6D_6).

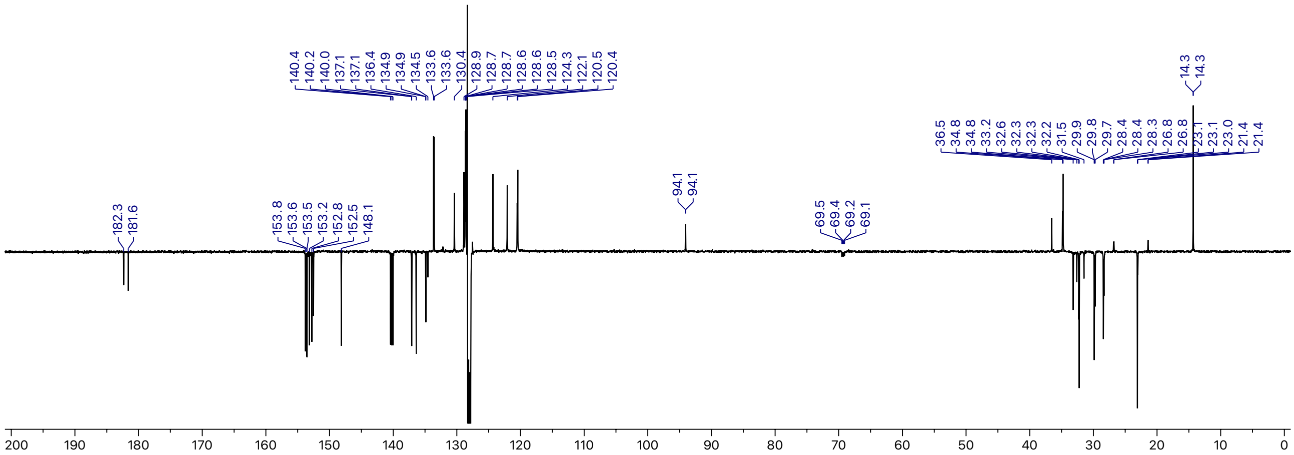

Figure S22. $^{13}C\{^1H\}$ APT NMR spectrum of [Rh(JEKphos)(acac)] (126 MHz, C_6D_6).

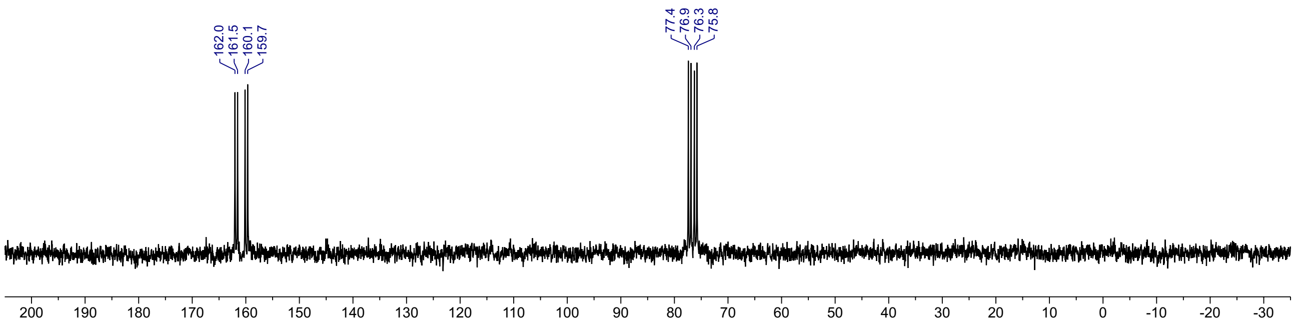

Figure S23. $^{31}P\{^1H\}$ NMR spectrum of [Rh(JEKphos)(acac)] (162 MHz, C_6D_6).

1.7 Preparation of [Rh((S_{ax},S,S)-bobphos)(acac)]

A solution of (S_{ax},S,S)-bobphos (0.203 g, 0.311 mmol) in toluene (15 mL) was added to a flask charged with [Rh(CO)$_2$(acac)] (0.0804 g, 0.312 mmol) and the resulting yellow suspension stirred at room temperature for 2 h. Volatiles were removed *in vacuo* to give the product as an orange solid, which was washed with hexane (5 mL) at –78 °C and dried. Yield: 0.193 g (0.226 mmol, 73%). Single crystals suitable for X-ray diffraction were obtained from a hexane solution at −30 °C.

^{1}H NMR (500 MHz, C_6D_6): δ 7.75 (d, $^{3}J_{HH}$ = 7.9, 2H, *o*-Ph), 7.49 (d, $^{3}J_{HH}$ = 7.8, 2H, *o*-Ph), 7.28 (t, $^{3}J_{HH}$ = 7.7, 2H, *m*-Ph), 7.26 (s, 1H, ArH), 7.24 (t, $^{3}J_{HH}$ = 7.7, 2H, *m*-Ph), 7.18 (s, 1H, ArH), 7.11 (t, $^{3}J_{HH}$ = 7.3, 1H, *p*-Ph), 7.08 (t, $^{3}J_{HH}$ = 7.5, 1H, *p*-Ph), 5.28 (s, 1H, C(O)CH), 4.47 (app td, J = 10.9, $^{3}J_{HH}$ = 6.7, 1H, PCH), 3.26–3.38 (m, 2H, 2×PCH$_2$), 3.01 (app td, J = 11.8, $^{3}J_{HH}$ = 7.1, 1H, PCH), 2.22–2.36 (m, 1H, PCHC$\underline{H_2}$), 2.15 (s, 3H, Ar$\underline{Me}$), 2.08–2.21 (obs m, 2H, 2×PCHC$\underline{H_2}$), 2.04 (s, 3H, Ar$\underline{Me}$), 1.95 (s, 3H, C(O)CH$_3$), 1.86 (s, 3H, Ar$\underline{Me}$), 1.74 (s, 9H, *t*Bu), 1.63–1.76 (obs m, 1H, PCHC$\underline{H_2}$), 1.66 (s, 3H, Ar$\underline{Me}$), 1.42 (s, 3H, C(O)CH$_3$), 1.34 (s, 9H, *t*Bu).

^{1}H{^{31}P} NMR (500 MHz, C_6D_6, selected data): δ 4.47 (dd, $^{3}J_{HH}$ = 12.3, 6.9, 1H, PCH), 3.34 (d, $^{2}J_{HH}$ = 11.9, 1H, PCH$_2$), 3.31 (d, $^{2}J_{HH}$ = 11.9, 1H, PCH$_2$), 3.02 (dd, $^{3}J_{HH}$ = 11.6, 7.1, 1H, PCH), 2.29 (app qd, J = 12.3, $^{3}J_{HH}$ = 5.4, 1H, PCHC$\underline{H_2}$), 1.70 (obs app qd, J = 12.6, $^{3}J_{HH}$ = 5.7, 1H, PCHC$\underline{H_2}$).

^{13}C{^{1}H} NMR (126 MHz, C_6D_6): δ 186.0 (s, C=O), 183.9 (s, C=O), 146.5 (d, $^{2}J_{PC}$ = 16, ArO), 146.1 (d, $^{2}J_{PC}$ = 4, ArO), 141.3 (d, $^{2}J_{PC}$ = 3, *i*-Ph), 138.4 (d, $^{3}J_{PC}$ = 4, $\underline{Ar}$*t*Bu), 137.9 (d, $^{2}J_{PC}$ = 4, *i*-Ph), 137.3 (d, $^{3}J_{PC}$ = 2, $\underline{Ar}$*t*Bu), 134.9 (s, $\underline{Ar}$Me), 134.6 (s, $\underline{Ar}$Me), 131.79 (s, $\underline{Ar}$Me), 131.74 (s, $\underline{Ar}$Me), 131.2 (d, $^{3}J_{PC}$ = 2, Ar–Ar), 130.9 (s, $^{3}J_{PC}$ = 2, Ar–Ar), 130.0 (d, $^{3}J_{PC}$ = 6, *o*-Ph), 128.9–129.0 (m, *m*-Ph+ArH), 127.7–128.4 (obs, *o*-Ph+*m*-Ph+ArH), 126.81 (d, $^{5}J_{PC}$ = 2, *p*-Ph), 126.74 (d, $^{5}J_{PC}$ = 2, *p*-Ph), 99.7 (d, $^{3}J_{RhC}$ = 2, C(O)$\underline{C}$H), 64.4 (app t, J = 19, PCH$_2$), 50.6 (d, $^{1}J_{PC}$ = 21, PCH), 45.7 (d, $^{1}J_{PC}$ = 18, PCH), 35.7 (d, $^{2}J_{PC}$ = 5, PCH$\underline{C}$H$_2$), 35.6 (s, *t*Bu{C}), 34.8 (s, *t*Bu{C}), 33.3 (s, *t*Bu{CH$_3$}), 32.1 (s, *t*Bu{CH$_3$}), 31.1 (br, PCH$\underline{C}$H$_2$), 28.2 (d, $^{4}J_{PC}$ = 8, C(O)$\underline{C}$H$_3$), 27.5 (d, $^{4}J_{PC}$ = 5, C(O)$\underline{C}$H$_3$), 20.4 (s, Ar$\underline{Me}$), 20.3 (s, Ar$\underline{Me}$), 16.8 (s, Ar$\underline{Me}$), 16.7 (s, Ar$\underline{Me}$).

^{31}P{^{1}H} NMR (202 MHz, C_6D_6): δ 164.8 (dd, $^{1}J_{RhP}$ = 318, $^{2}J_{PP}$ = 71, 1P, phosphite), 113.3 (dd, $^{1}J_{RhP}$ = 192, $^{2}J_{PP}$ = 71, 1P, phosphine).

Anal. Calcd for $C_{46}H_{57}O_5P_2Rh$ (854.81 g·mol^{-1}): C, 64.63; H, 6.72; Found: C, 64.84; H, 6.71.

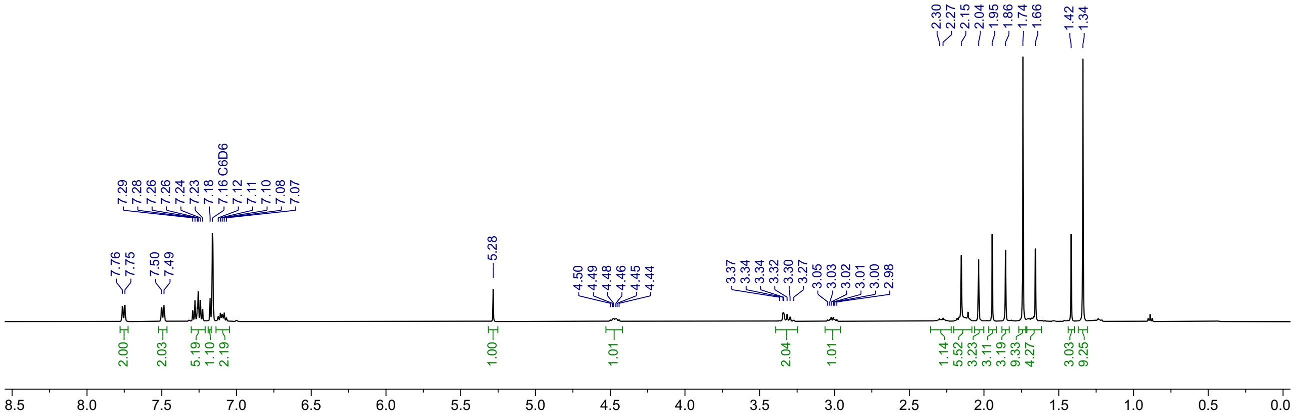

Figure S24. ^{1}H NMR spectrum of [Rh((*S*$_{ax}$,*S*,*S*)-bobphos)(acac)] (500 MHz, C_6D_6).

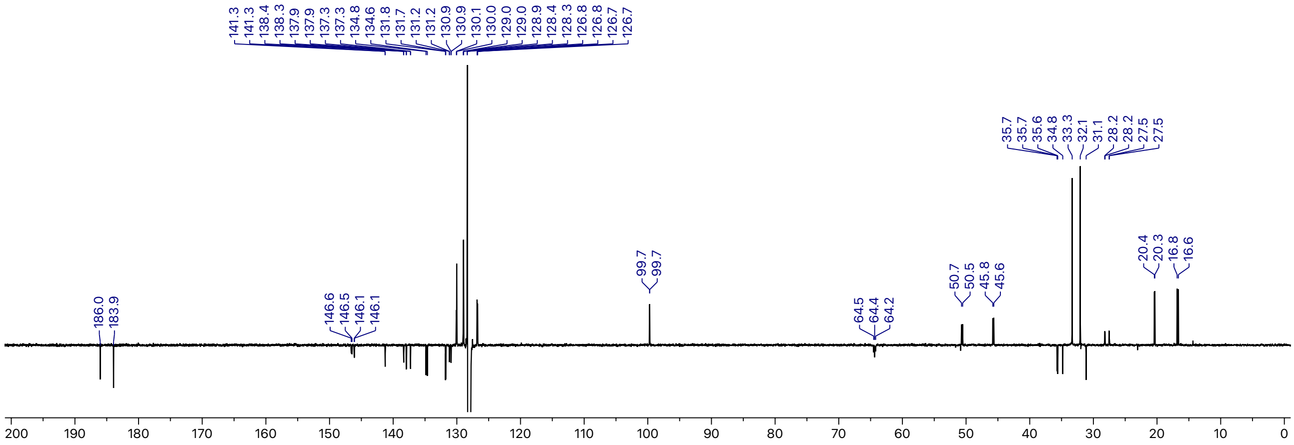

Figure S25. ^{13}C{^{1}H} APT NMR spectrum of [Rh((*S*$_{ax}$,*S*,*S*)-bobphos)(acac)] (126 MHz, C_6D_6).

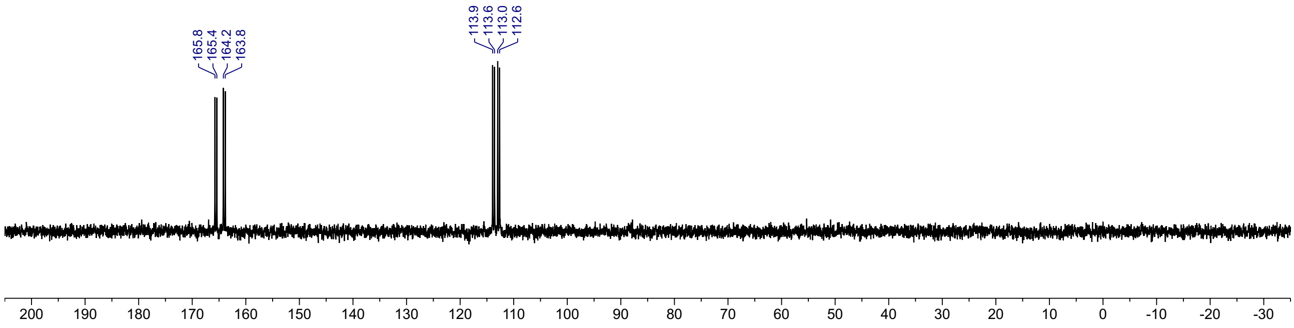

Figure S26. ^{31}P{^{1}H} NMR spectrum of [Rh((*S*$_{ax}$,*S*,*S*)-bobphos)(acac)] (202 MHz, C_6D_6).

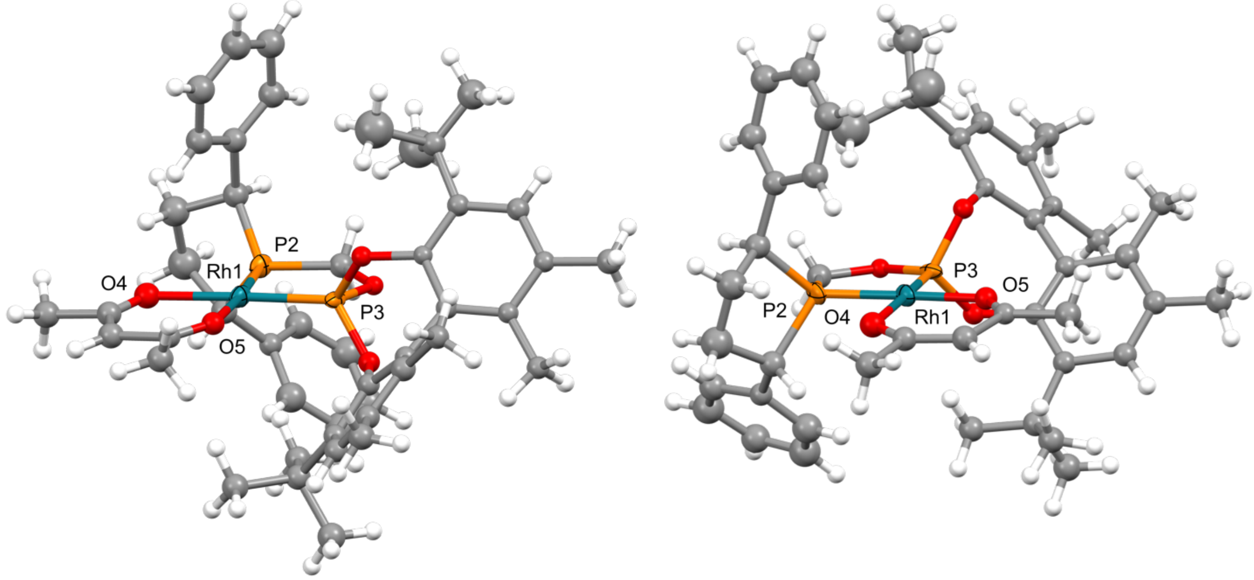

Figure S27. Solid-state structure of [Rh((S_{ax},S,S)-bobphos)(acac)]. Two perspectives of one of the two unique but structurally similar molecules shown (Z' = 2) with thermal ellipsoids drawn at 30% probably. Due to the low quality of the data collected (R_{int} = 0.1465), only heavy atoms were refined anisotropically. Structure provided to confirm bond connectivity only.

1.8 Preparation of [Rh(dppe)(acac)]

A solution of dppe (0.2046 g, 0.5135 mmol) in toluene (10 mL) was added to a flask charged with [Rh(CO)$_2$(acac)] (0.1325 g, 0.5135 mmol) and the resulting yellow suspension stirred at room temperature for 2 h. Volatiles were removed *in vacuo* to give a yellow residue, which was washed with hexane (10 mL) and dried. The product was extracted into THF (10 mL) and isolated as a brown solid upon removal of volatiles and trituration with a small amount of toluene. Yield: 0.2164 g (0.3586 mmol, 70%). Single crystals suitable for X-ray diffraction were obtained by slow diffusion of hexane into a C_6H_5F solution at room temperature. ^{31}P NMR data is consistent with the literature (δ_{31P} 70.0, $^1J_{RhP}$ = 194 Hz).[12]

^{1}H NMR (500 MHz, C_6D_6): δ 8.00–8.07 (m, 8H, *o*-Ph), 7.02–7.11 (m, 12H, *m*,*p*-Ph), 5.41 (s, 1H, CH), 1.84 (s, 6H, CH_3), 1.83 (app d, J = 17.1, 4H, PCH_2).

^{13}C{^{1}H} NMR (126 MHz, C_6D_6,): δ 185.0 (s, C=O), 137.0–138.0 (m, *i*-Ph), 133.4 (vt, J_{PC} = 11, *o*-Ph), 129.4 (br, *p*-Ph), 128.2 (obs, *m*-Ph), 99.8 (d, $^3J_{RhC}$ = 2, C(O)CH), 28.1 (vt, J_{PC} = 6, C(O)CH_3), 27.8 (vtd, J_{PC} = 51, $^2J_{RhC}$ = 4, PCH_2).

^{31}P{^{1}H} NMR (162 MHz, C_6D_6,): δ 70.2 (d, $^1J_{RhP}$ = 193)

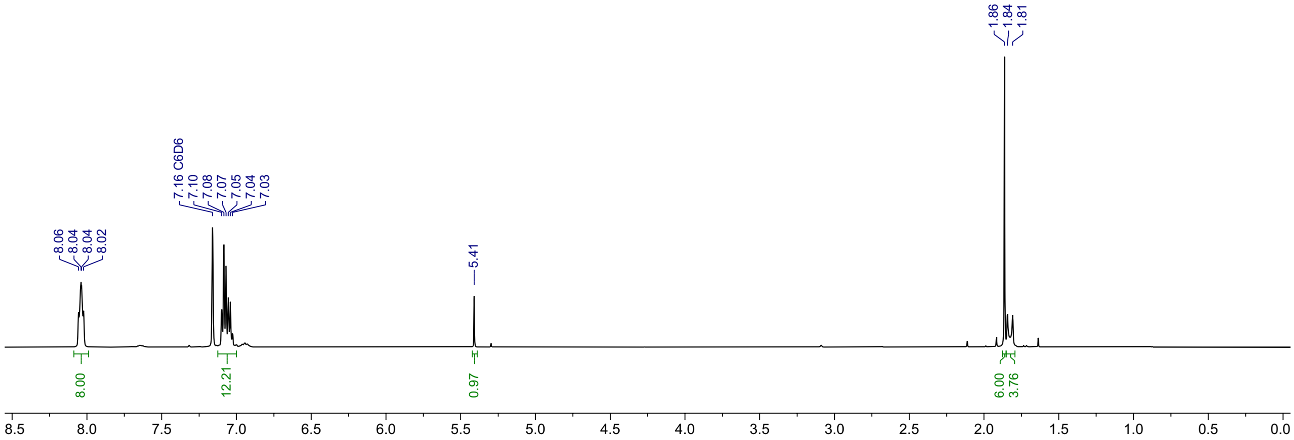

Figure S28. ^{1}H NMR spectrum of [Rh(dppe)(acac)] (500 MHz, C_6D_6).

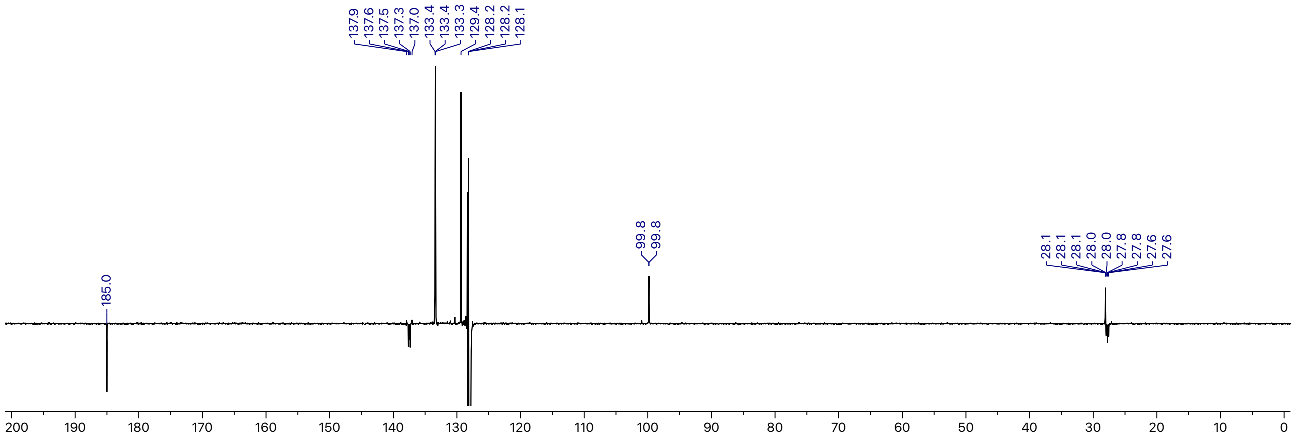

Figure S29. ^{13}C{^{1}H} APT NMR spectrum of [Rh(dppe)(acac)] (126 MHz, C_6D_6).

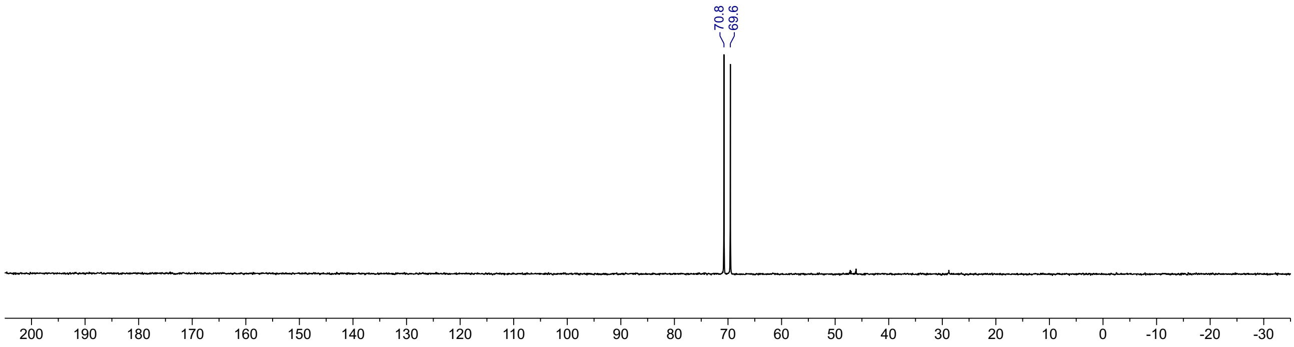

Figure S30. ^{31}P{^{1}H} NMR spectrum of [Rh(dppe)(acac)] (162 MHz, C_6D_6).

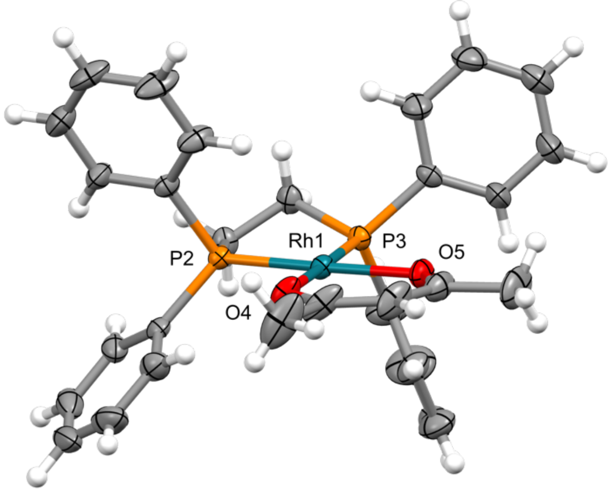

Figure S31. Solid-state structure of [Rh(dppe)(acac)]. Thermal ellipsoids drawn at 50% probably. Selected data: Rh1–P2, 2.1810(10) Å; Rh1–P3, 2.1786(12) Å; Rh1–O4, 2.080(3) Å; Rh1–O5, 2.072(3) Å; P2–Rh1–P3, 84.85(4)°; O4–Rh1–O5, 88.80(14)°.

2 Hydroformylation reactions

2.1 General methods

All manipulations were performed under an atmosphere of inert gas using Schlenk (dinitrogen) and glove box (argon) techniques unless otherwise stated. Glassware was oven dried at 200 °C overnight and flame-dried under vacuum prior to use. Molecular sieves were activated by heating at 300 °C *in vacuo*. Toluene was purified from a solvent purification system and stored under dinitrogen over 3 Å molecular sieves. 1-hexene, 1-heptene and 1-octene were filtered through acidified Al_2O_3 under dinitrogen prior to use. NMR spectra for analysis of hydroformylation reaction mixtures were recorded on a Bruker 400 MHz spectrometer. NMR spectra for *in situ* analysis were recorded on a 500 MHz Bruker Avance Neo using a room temperature BBFO probe.

2.2 Hydroformylation reactions

All reactions were performed using a custom-built high-pressure autoclave equipped with a pressure gauge, injection port and addition funnel (Figure S32). Under an atmosphere of dinitrogen a solution of rhodium precatalyst (12.0 μmol) in toluene (1.5 mL) was added to the autoclave. The atmosphere was purged and subsequently pressurised with 10 bar of a 1:1 mixture of CO and H_2. The solution was heated at the chosen reaction temperature under 10 bar CO/H_2 for 30 mins to activate the catalyst. The addition funnel was loaded with either 1-hexene (0.60 mL, 4.8 mmol), 1-heptene (0.68 mL, 4.8 mmol) or 1-octene (0.75 mL, 4.8 mmol) in toluene (1 mL) under a dinitrogen atmosphere and purged with CO/H_2 gas. The alkene solution was added to the autoclave using an overpressure of syngas (20 bar), raising the pressure of the reaction chamber to 20 bar, and heated at the chosen temperature for the specified time. The autoclave was then cooled to room temperature, depressurised, and the reaction quenched by the addition of excess P(O*n*Bu)$_3$ (1 mL). An aliquot (0.6 mL) was analysed by ^{1}H NMR spectroscopy, with product distributions determined

by integration of ^{1}H NMR data collected in *proteo*-toluene. Selected data: δ 9.36 (t, $^{3}J_{HH}$ = 1.7 Hz, linear CHO), 9.28 (d, $^{3}J_{HH}$ = 1.8 Hz, branched CHO), 5.68 (ddt, $^{3}J_{HH}$ = 16.9, 10.1, 6.7, 1-alkene HC=CH_2), 5.24–5.40 (m, 2-alkene HC=CHMe).

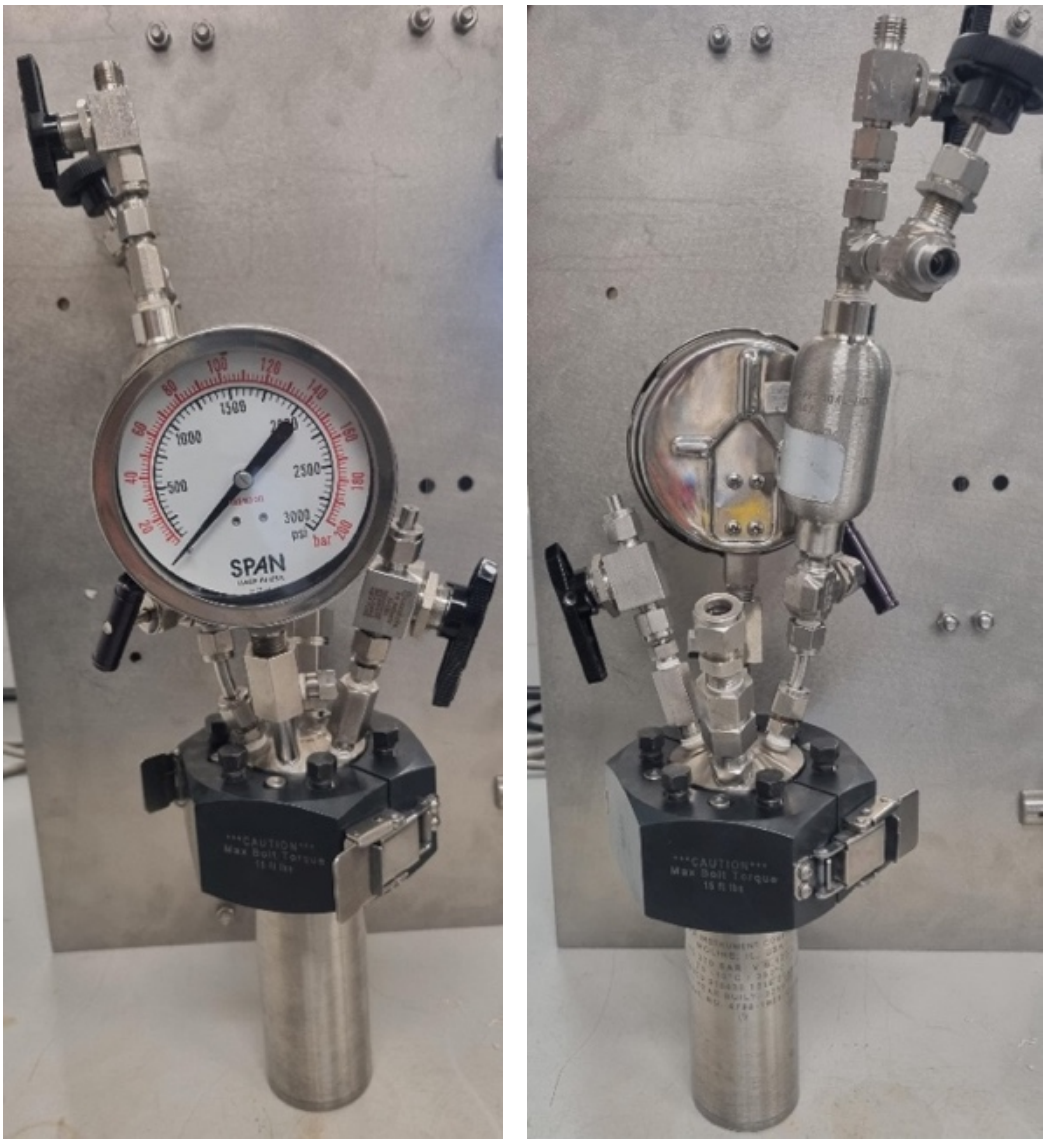

Figure S32. High-pressure autoclave used for hydroformylation reactions.

Table S1. Data from hydroformylation reactions

Entry	Substrate	*T* / °C	*t* / h	Conversion[a]	TOF / h^{-1}	*b/l*	Branched[b]	Isomerisation[b]
[Rh(JEKphos)(acac)]								
1	1-hexene	50	0.5	17.8%	142	1.06	48%	7.6%
2	1-hexene	50	0.5	17.5%	140	1.07	48%	8.0%
3	1-hexene	60	0.5	56.9%	455	0.92	45%	6.6%
4	1-hexene	60	0.5	55.9%	447	0.93	45%	7.0%
5	1-hexene	70	0.5	90.5%	724	0.85	43%[c]	5.4%
6	1-hexene	70	0.5	87.7%	702	0.86	43%[c]	6.1%
7	1-heptane	60	0.5	24.4%	195	2.51	64%	9.9%
8	1-heptane	60	0.5	30.0%	240	2.45	64%	9.6%
9	1-octene	60	0.5	21.8%	174	6.07	81%	6.0%
10	1-octene	60	0.5	22.8%	182	5.66	80%	5.4%
[Rh((S_{ax},*S*,*S*)-bobphos)(acac)]								
11	1-hexene	60	0.5	22.2%	178	2.30	67%	3.3%
12	1-hexene	60	0.5	16.5%	132	2.30	66%	5.4%
13	1-heptane	60	0.5	17.0%	136	2.22	65%	5.6%
14	1-heptane	60	0.5	14.1%	113	2.25	65%	5.8%
15	1-octene	60	0.5	19.7%	158	2.23	66%	4.4%
16	1-octene	60	0.5	19.5%	156	2.19	66%	4.3%
[Rh(dppe)(acac)]								
17	1-hexene	60	1.5	23.6%	63	0.41	28%	3.8%
18	1-hexene	60	1.5	29.5%	79	0.41	28%	4.7%
19	1-heptane	60	1.5	16.4%	44	0.46	30%	6.1%
20	1-heptane	60	1.5	24.6%	66	0.42	28%	5.0%
21	1-octene	60	1.5	29.8%	79	0.41	28%	4.7%
22	1-octene	60	1.5	25.9%	68	0.43	28%	5.3%

[a] 1-alkene into aldehyde. [b] percentage of internal aldehyde and alkene products. [c] trace quantities of 3-methylhexanal observed.

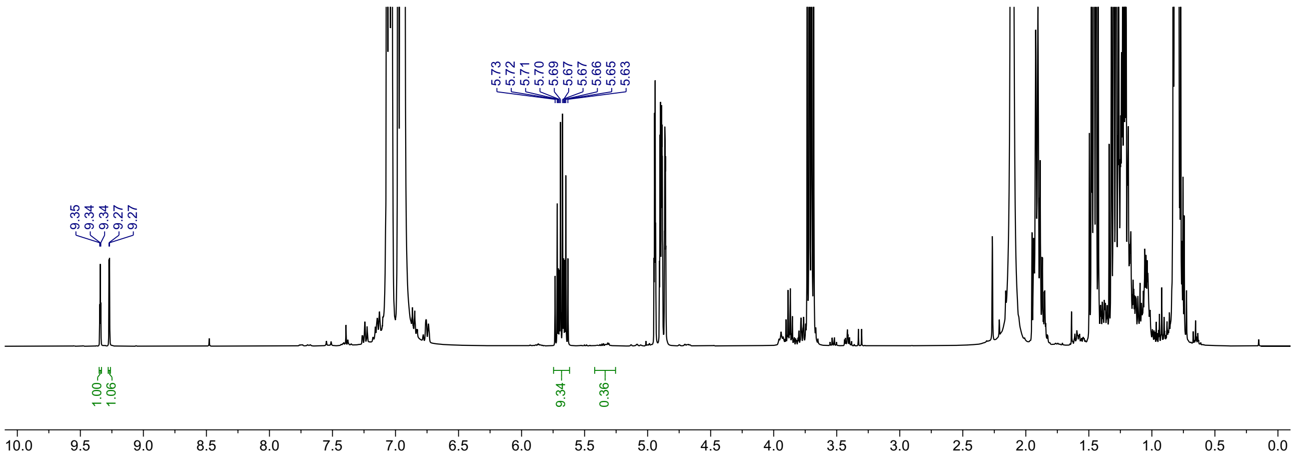

Figure S33. ^{1}H NMR spectrum for experiment 1.

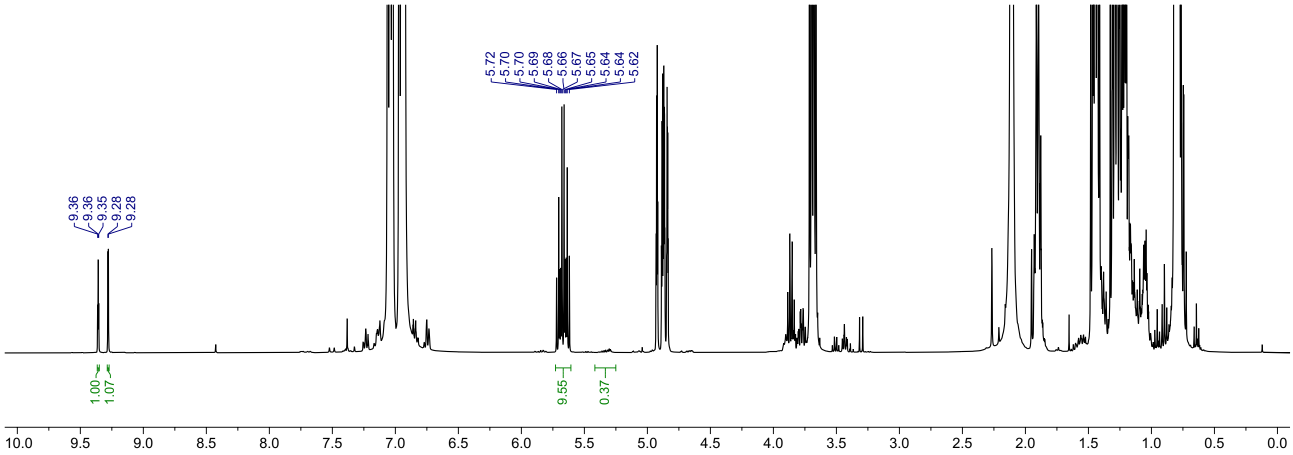

Figure S34. ^{1}H NMR spectrum for experiment 2.

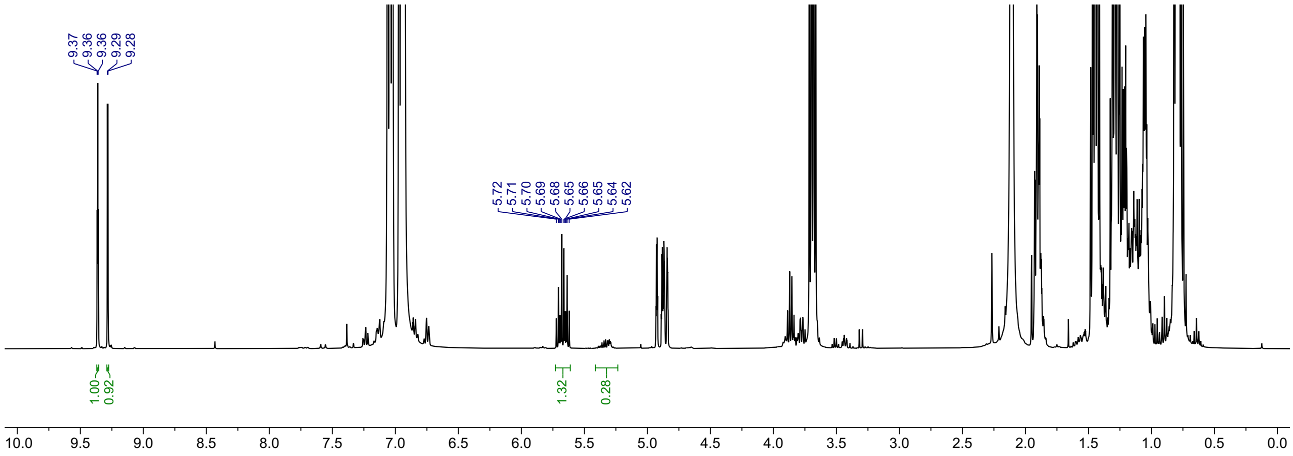

Figure S35. ^{1}H NMR spectrum for experiment 3.

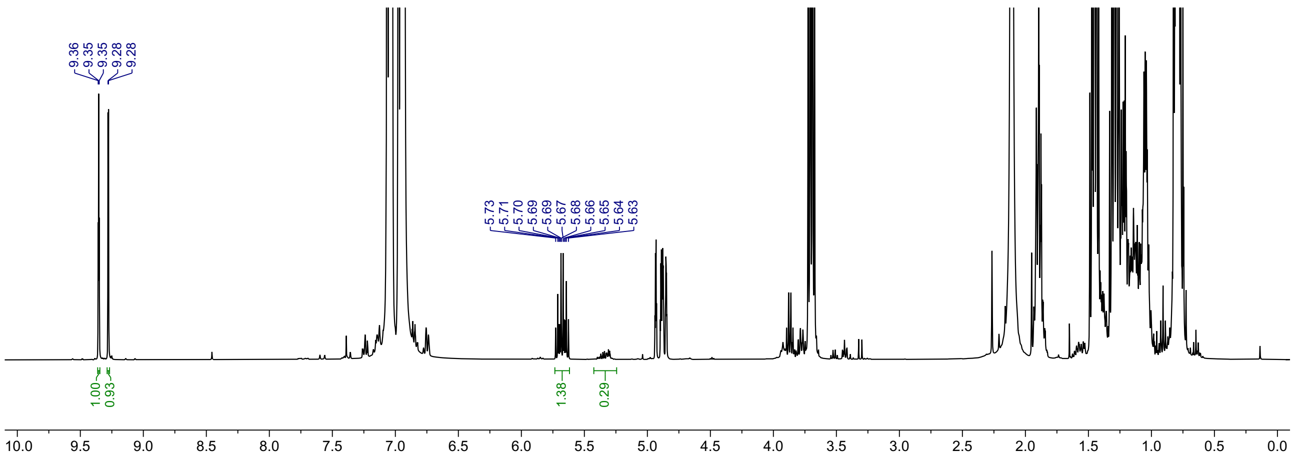

Figure S36. ^{1}H NMR spectrum for experiment 4.

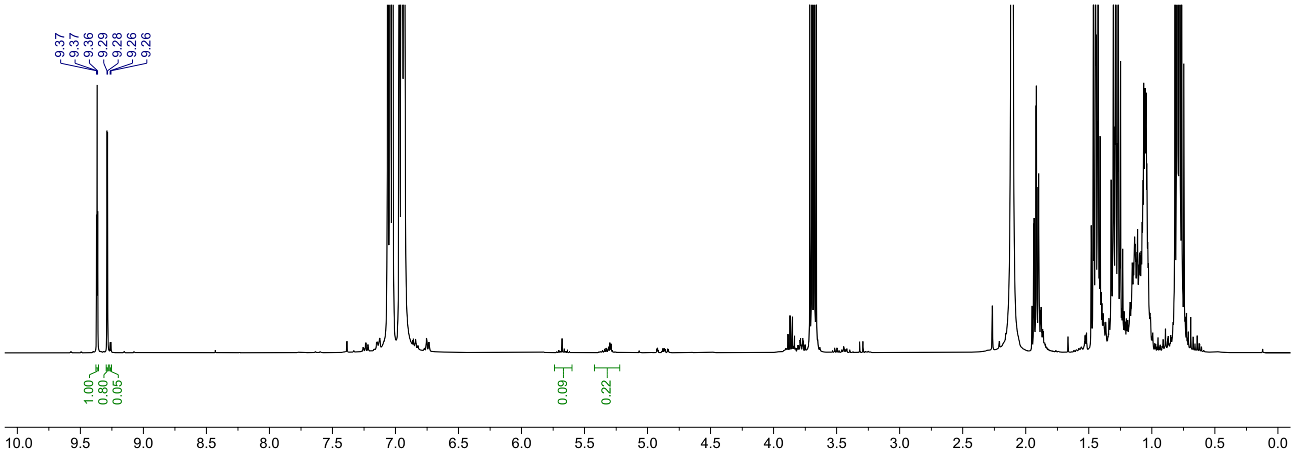

Figure S37. ^{1}H NMR spectrum for experiment 5.

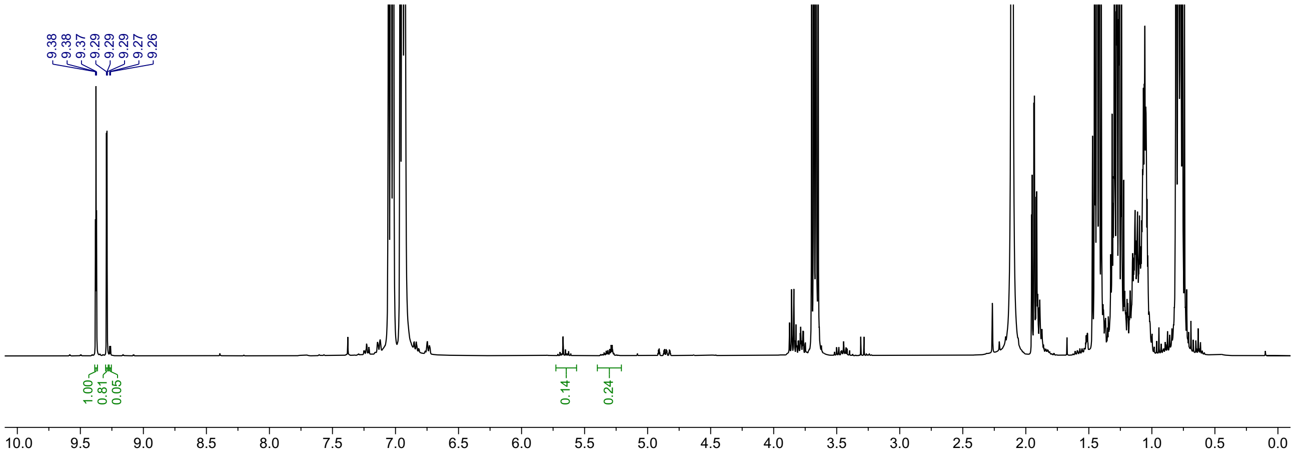

Figure S38. ^{1}H NMR spectrum for experiment 6.

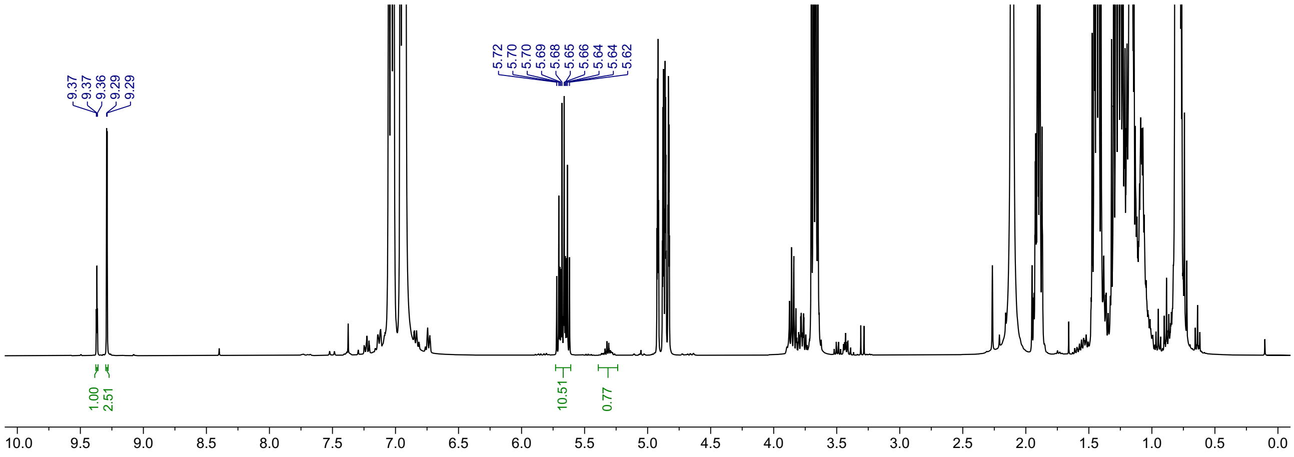

Figure S39. ^{1}H NMR spectrum for experiment 7.

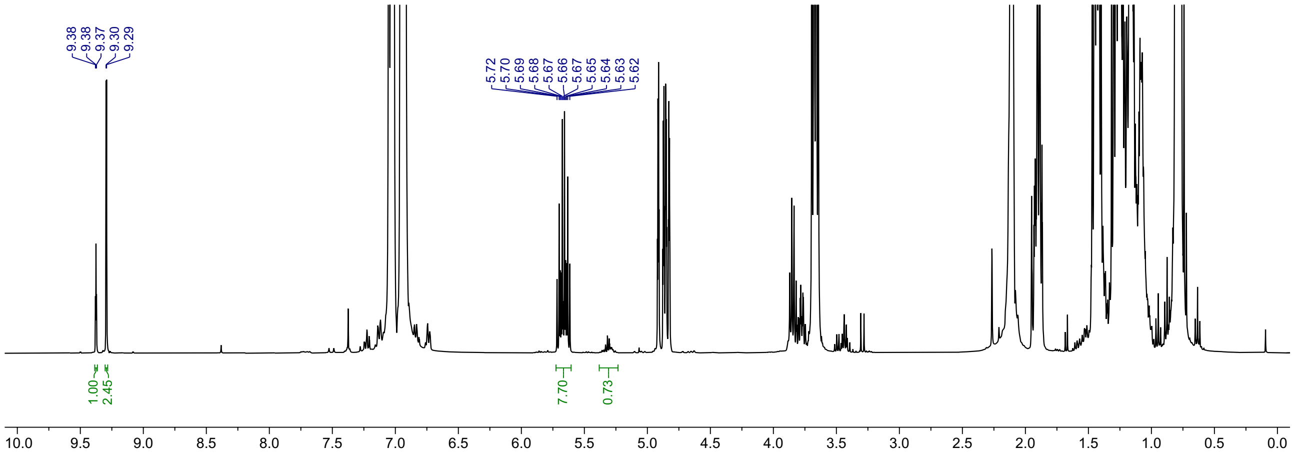

Figure S40. ^{1}H NMR spectrum for experiment 8.

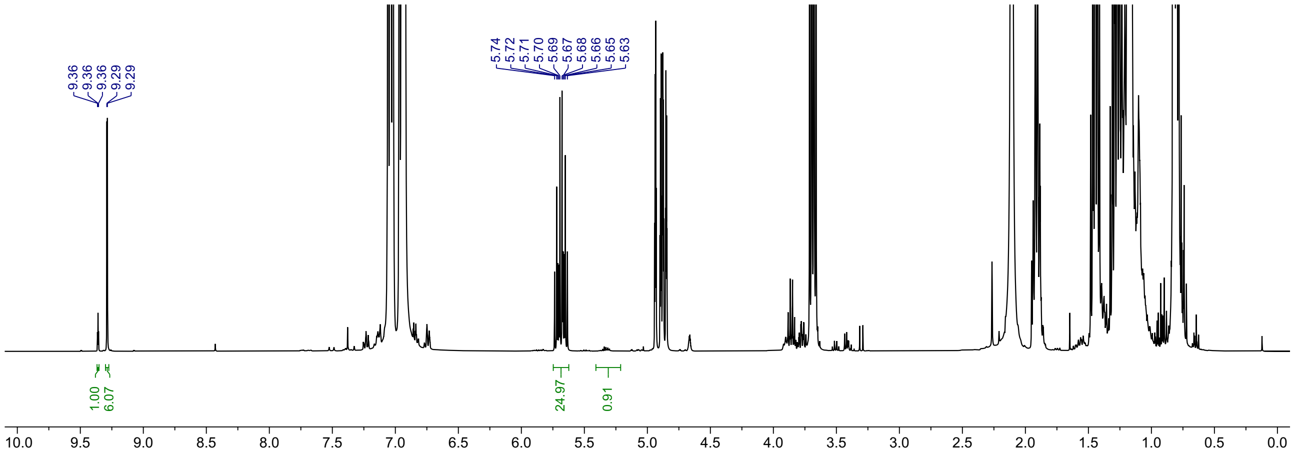

Figure S41. ^{1}H NMR spectrum for experiment 9.

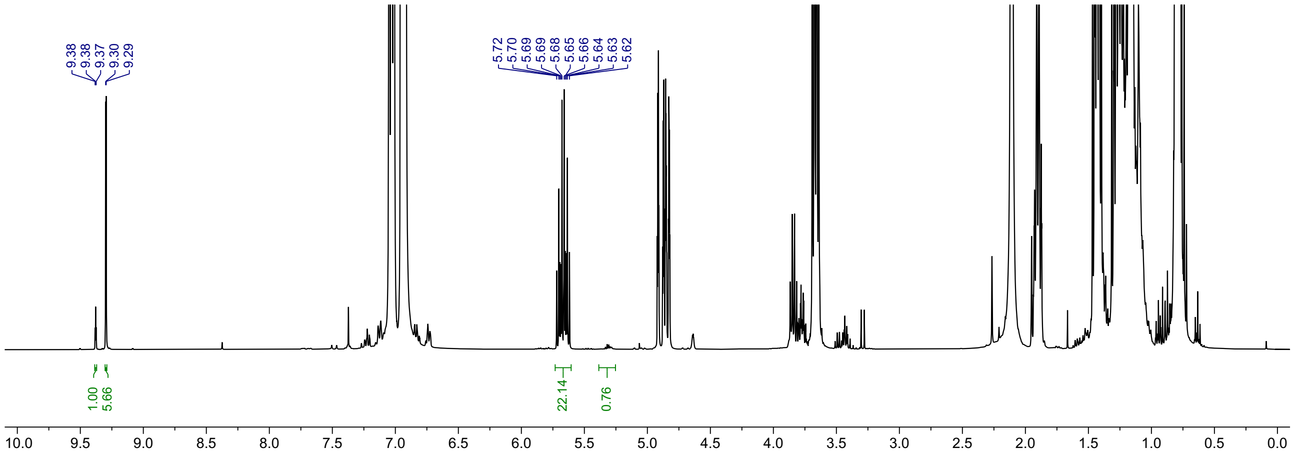

Figure S42. ^{1}H NMR spectrum for experiment 10.

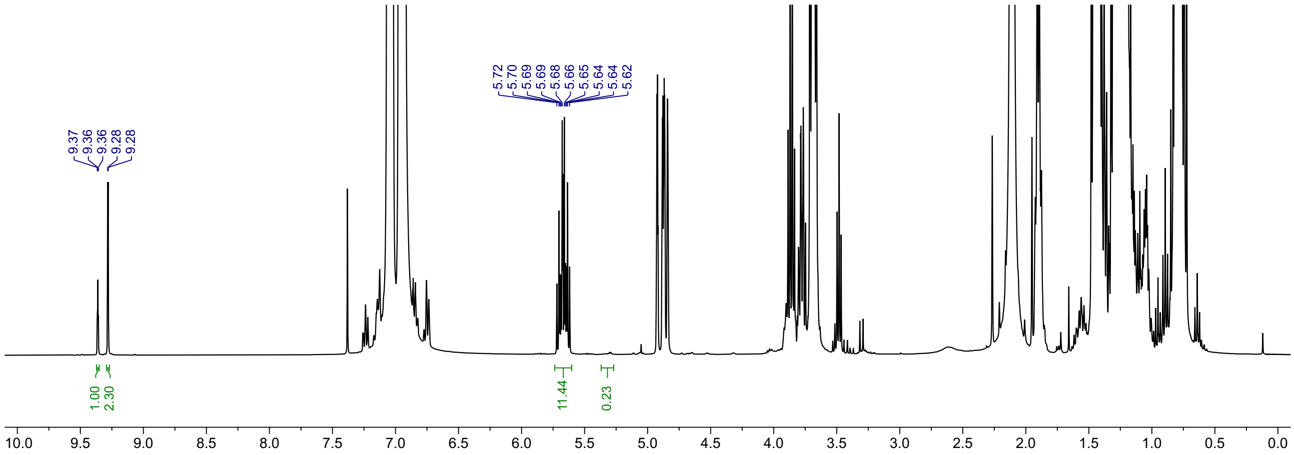

Figure S43. ^{1}H NMR spectrum for experiment 11.

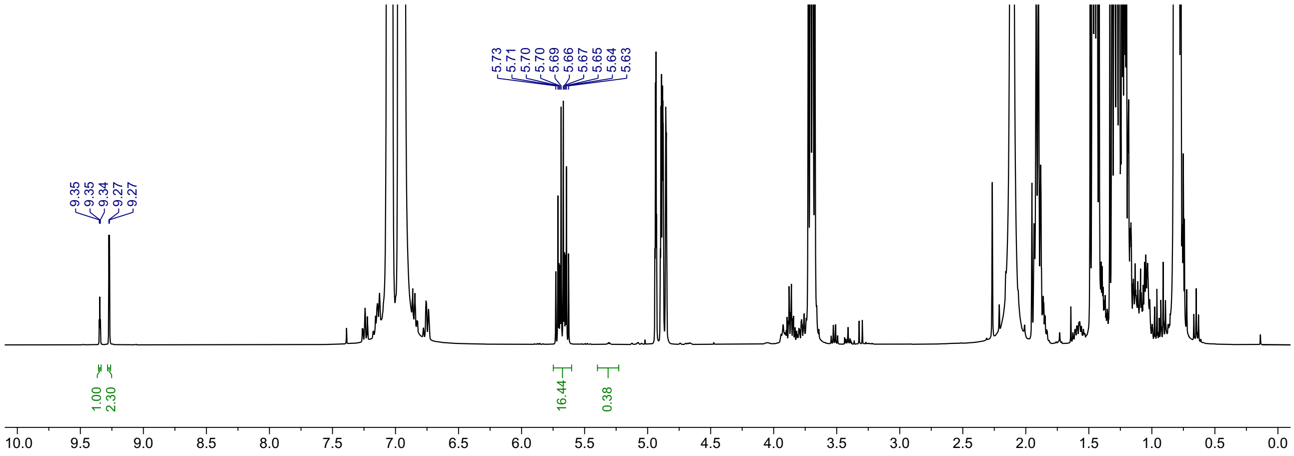

Figure S44. ^{1}H NMR spectrum for experiment 12.

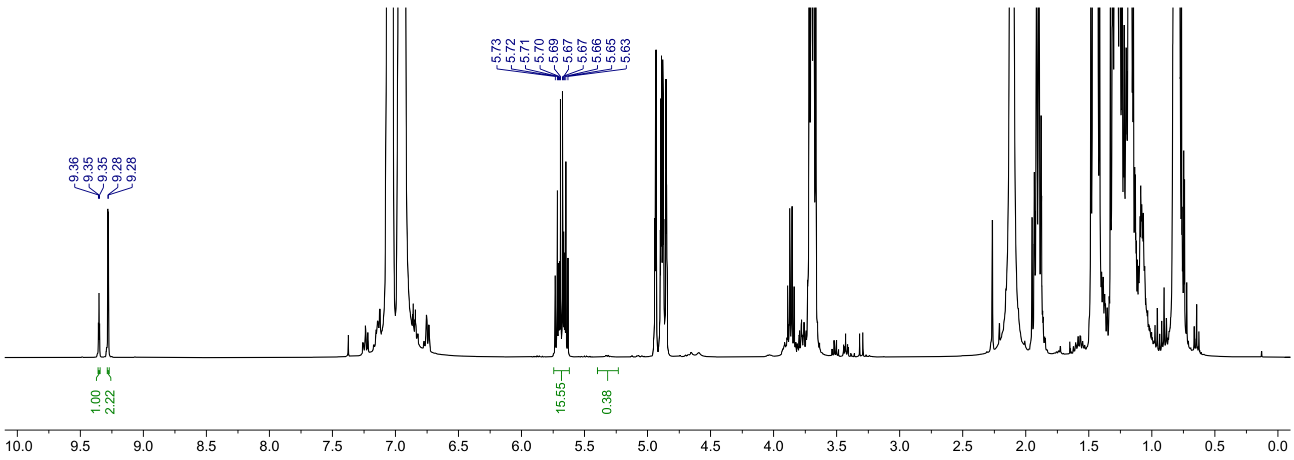

Figure S45. ^{1}H NMR spectrum for experiment 13.

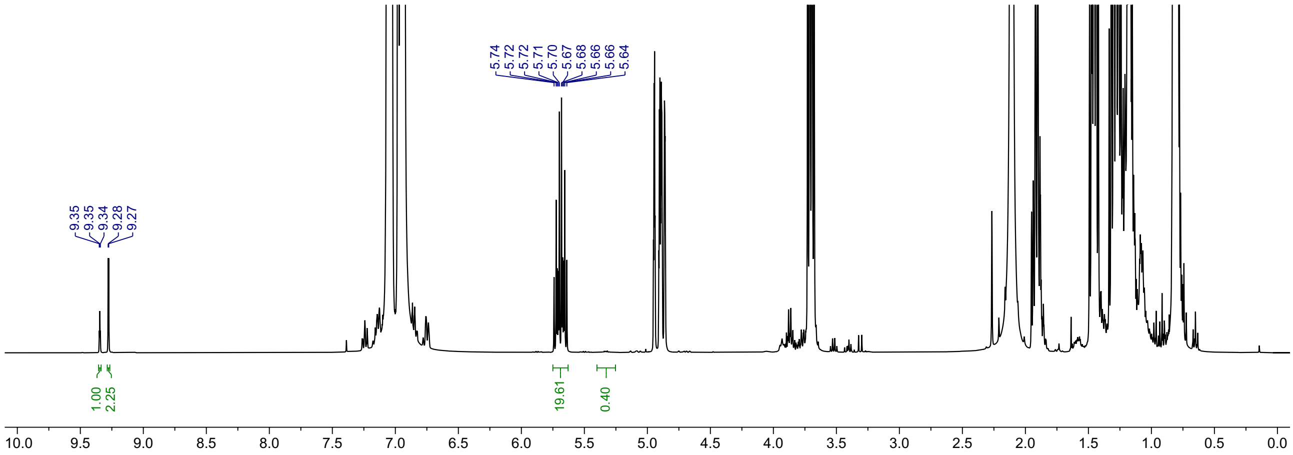

Figure S46. ^{1}H NMR spectrum for experiment 14.

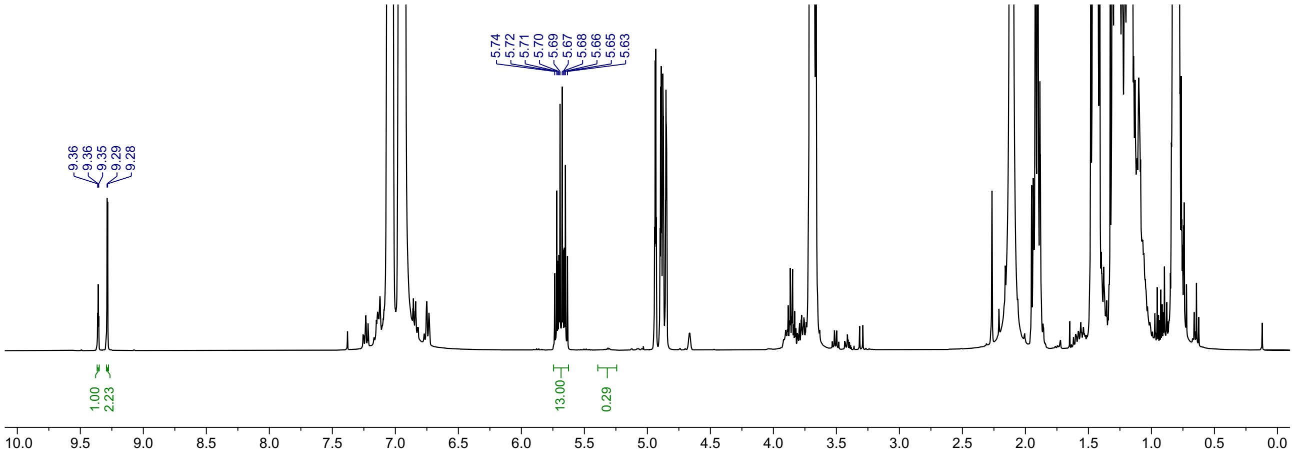

Figure S47. ^{1}H NMR spectrum for experiment 15.

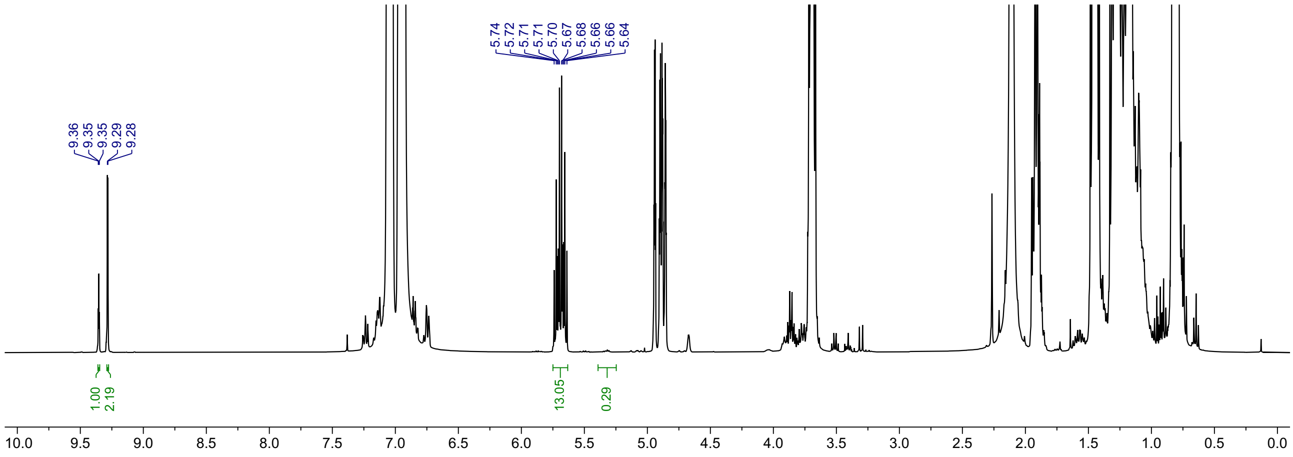

Figure S48. ^{1}H NMR spectrum for experiment 16.

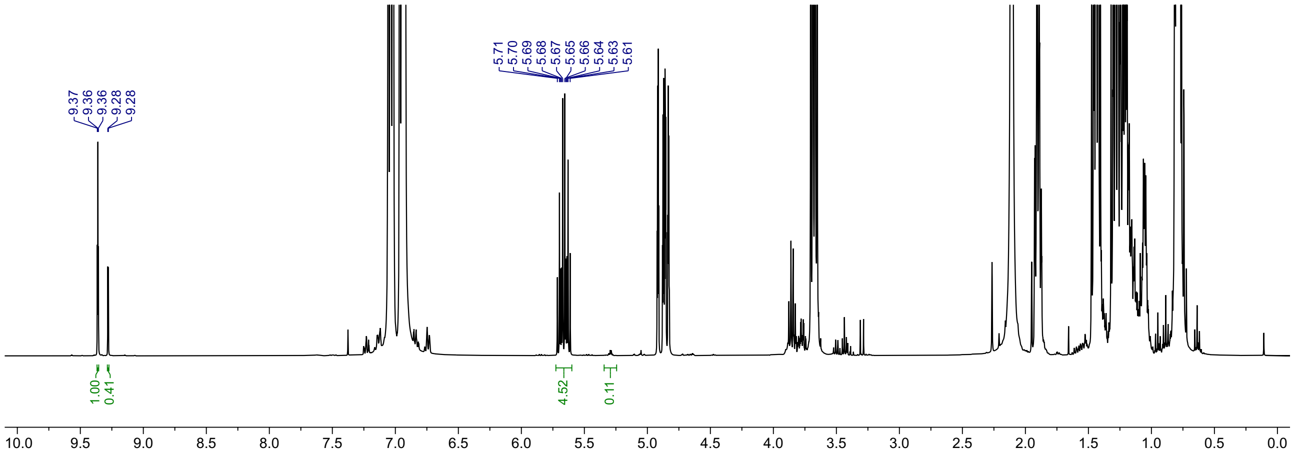

Figure S49. ^{1}H NMR spectrum for experiment 17.

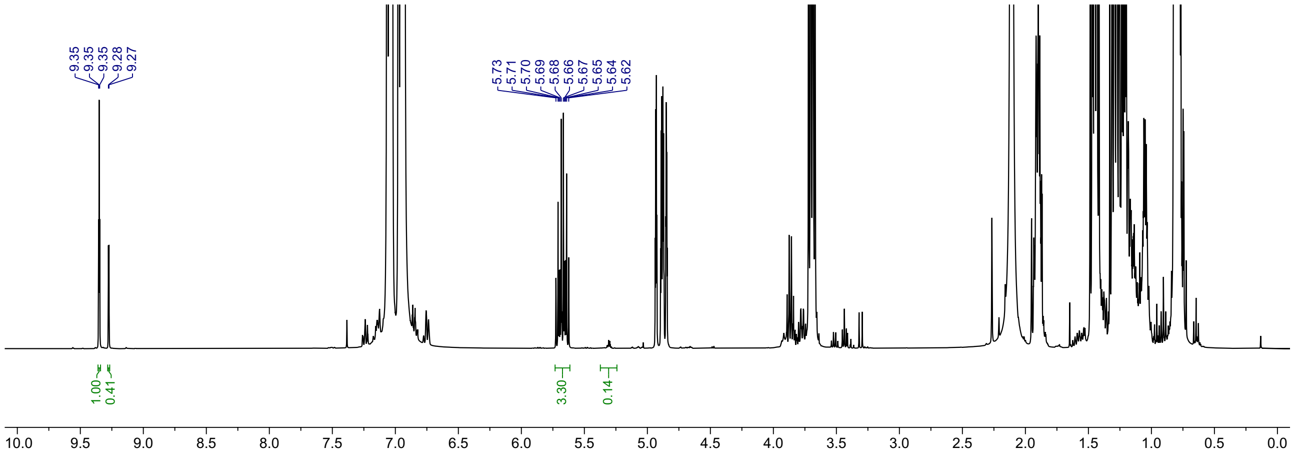

Figure S50. ^{1}H NMR spectrum for experiment 18.

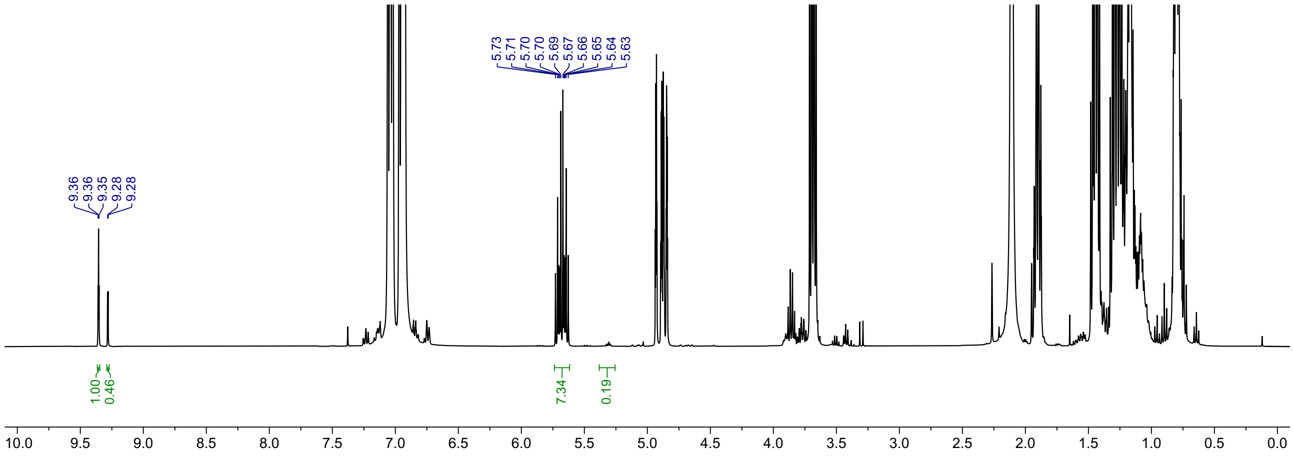

Figure S51. ^{1}H NMR spectrum for experiment 19.

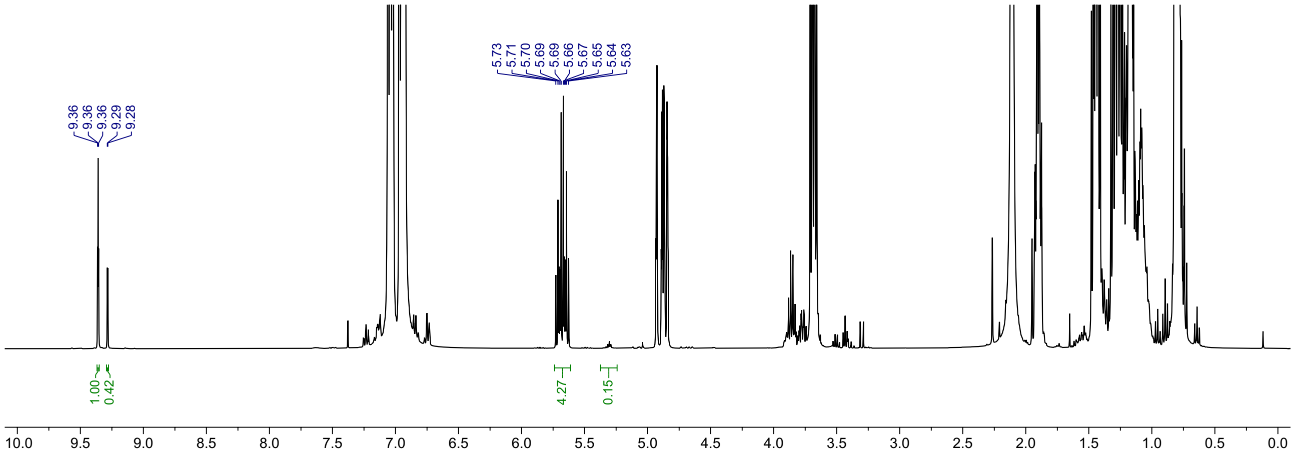

Figure S52. ^{1}H NMR spectrum for experiment 20.

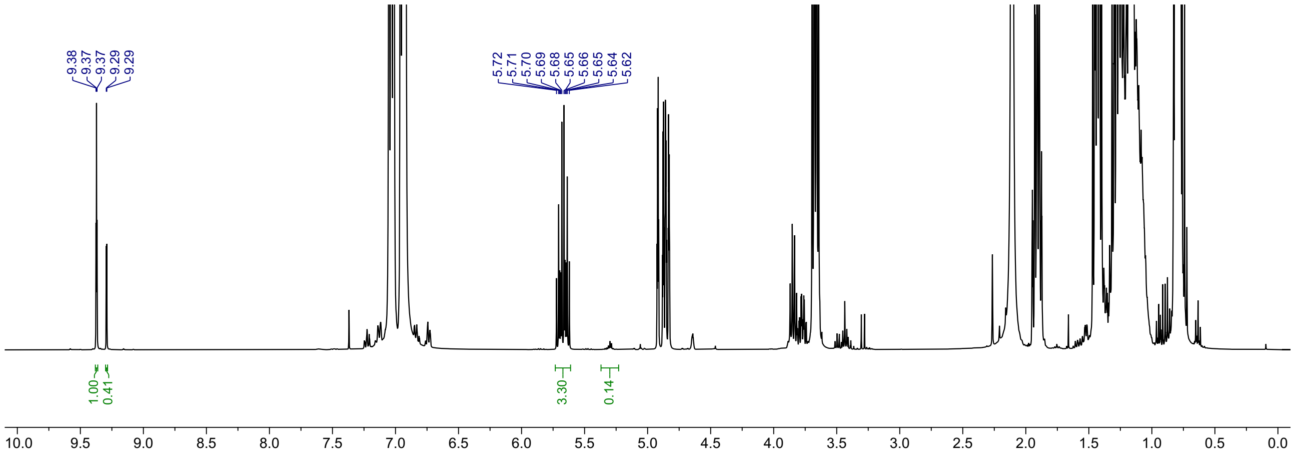

Figure S53. ^{1}H NMR spectrum for experiment 21.

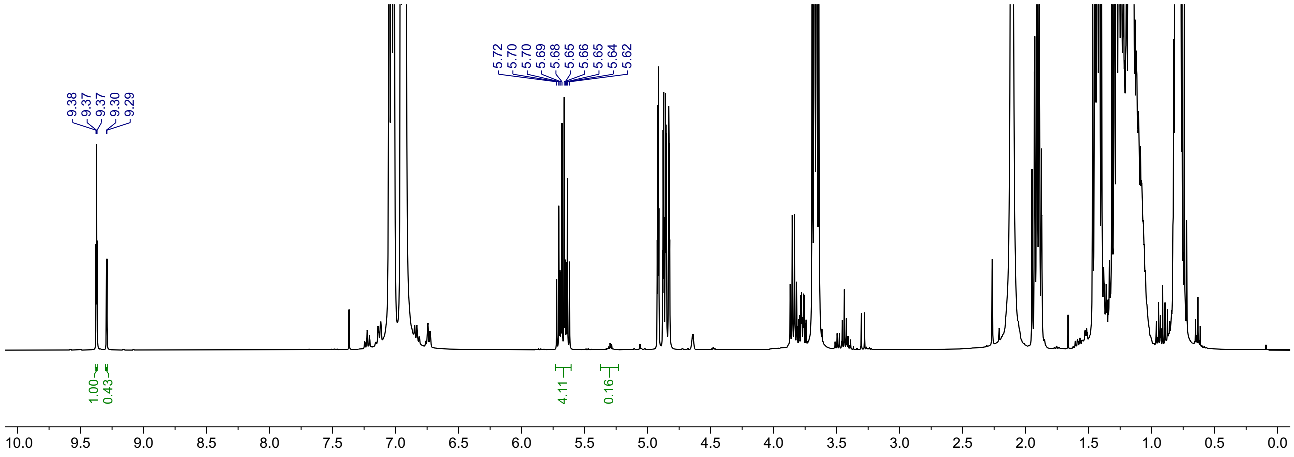

Figure S54. ^{1}H NMR spectrum for experiment 22.

2.3 Reaction of [Rh(JEKphos)(acac)] 5 with syngas

A solution of [Rh(JEKphos)(acac)] **5** (15 mg, 9.1 µmol) in toluene (1.5 mL) was stirred under 1:1 H_2/CO (20 bar) in a 10 mL autoclave for two days at room temperature. The autoclave was depressurised and a 0.5 mL aliquot was transferred to a J. Young valve NMR tube under a N_2 atmosphere. Analysis by NMR spectroscopy indicated formation of a unique organometallic

derivative, which was assigned to the trigonal pyramidal complex [Rh(JEKphos)H(CO)$_2$] **6** with the hydride and phosphine are in the axial positions.

^{1}H NMR (500 MHz, toluene, selected data): δ –9.99 (ddd, $^2J_{PH}$ = 108, 22, $^1J_{RhH}$ = 7, RhH).
^{1}H{^{31}P@181.1} NMR (500 MHz, toluene, selected data): δ –9.99 (dd, $^2J_{PH}$ = 108, $^1J_{RhH}$ = 7, RhH).
^{1}H{^{31}P@68.6} NMR (500 MHz, toluene, selected data): δ –9.99 (dd, $^2J_{PH}$ = 22, $^1J_{RhH}$ = 7, RhH).
^{31}P{^{1}H} NMR (202 MHz, toluene, selected data): δ 181.1 (dd, $^1J_{RhP}$ = 225, $^2J_{PP}$ = 43, 1P, phosphite), 68.6 (dd, $^1J_{RhP}$ = 98, $^2J_{PP}$ = 43, 1P, phosphine).

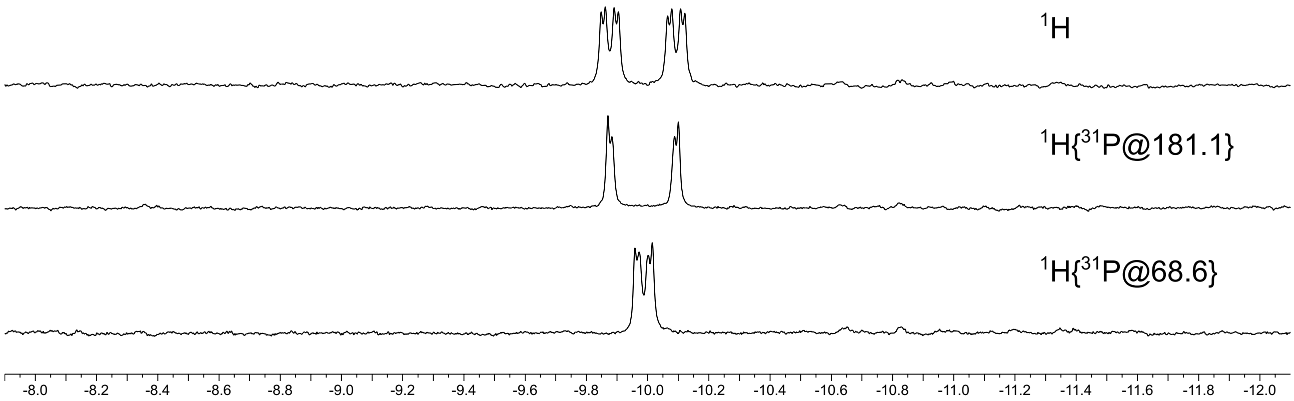

Figure S55. ^{1}H NMR spectra of [Rh(JEKphos)H(CO)$_2$] (500 MHz, toluene).

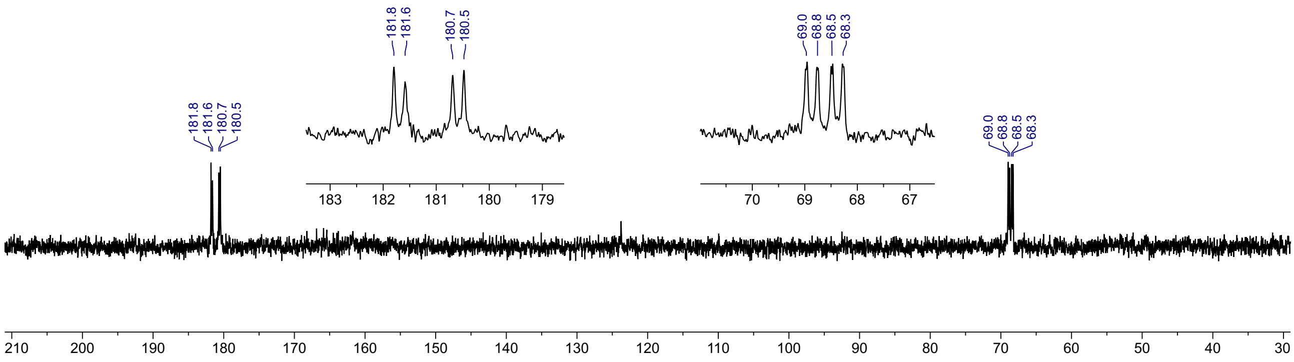

Figure S56. ^{31}P{^{1}H} NMR spectrum of [Rh(JEKphos)H(CO)$_2$] (202 MHz, toluene).

2.4 Reaction of [Rh(JEKphos)H(CO)$_2$] 6 generated *in situ* with CO/C_2H_4

A solution of [Rh(JEKphos)H(CO)$_2$] **6** (10 µmol) in d_8-toluene (1.0 mL) was prepared in a similar manner to that described above. A 0.5 mL aliquot was transferred into a J. Young valve NMR tube and placed under CO (1 bar). The tube was further pressurised with ethylene (2 bar) to a total pressure of 3 bar and the allowed to stand for 5 minutes at room. Analysis by NMR spectroscopy indicated conversation of **6** into trigonal pyramidal complex [Rh(JEKphos)(COEt)(CO)$_2$] **7**, which was

the major organometallic derivative present in solution. Formation and assignment of the acyl ligand was confirmed using a combination of COSY and HMBC experiments.

^{1}H NMR (500 MHz, d_8-toluene, selected data): δ –1.06 (br, 2H, RhCOC$\underline{H}_2$CH$_3$), –3.33 (br, 3H, RhCOCH$_2$C$\underline{H}_3$).
^{13}C{^{1}H} NMR (126 MHz, d_8-toluene, selected data): δ 232.0 (dd, $^{1}J_{RhC}$ = 93, $^{2}J_{PC}$ = 22, Rh$\underline{C}$OCH$_2$CH$_3$),
^{31}P{^{1}H} NMR (202 MHz, d_8-toluene, selected data): δ 171.2 (d, $^{1}J_{RhP}$ = 238, 1P, phosphite), 52.6 (vbr, fwhm = 150 Hz, 1P, phosphine).

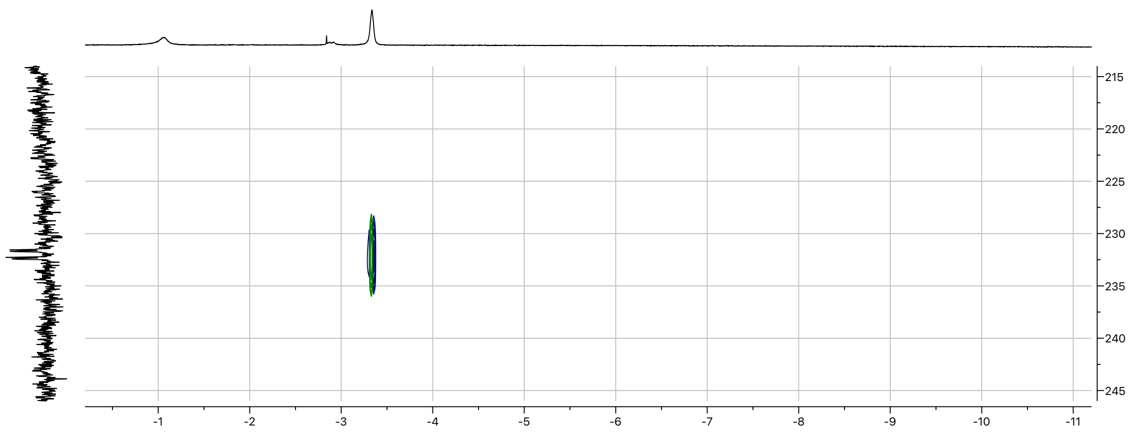

Figure S57. ^{1}H-^{13}C HMBC spectrum of [Rh(JEKphos)(COEt)(CO)$_2$] (d_8-toluene).

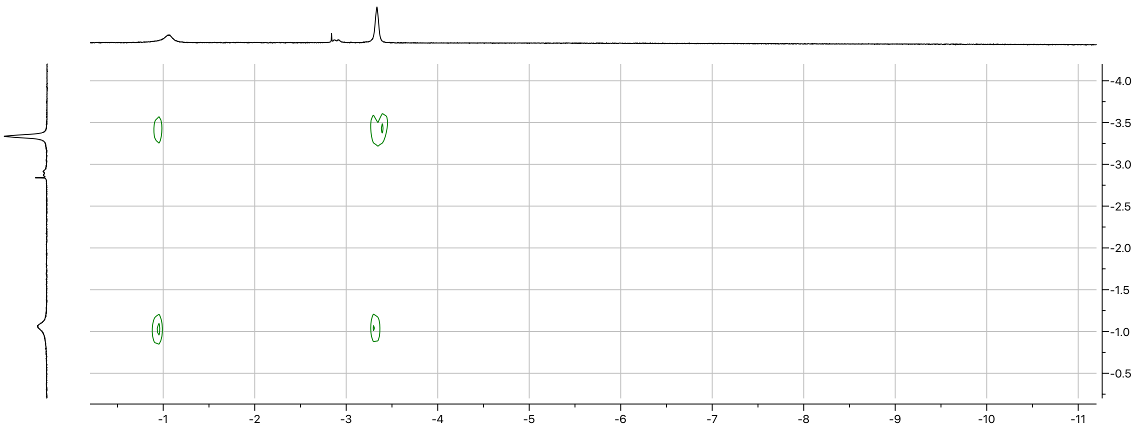

Figure S58. ^{1}H-^{1}H COSY spectrum of [Rh(JEKphos)(COEt)(CO)$_2$] (500 MHz, d_8-toluene).

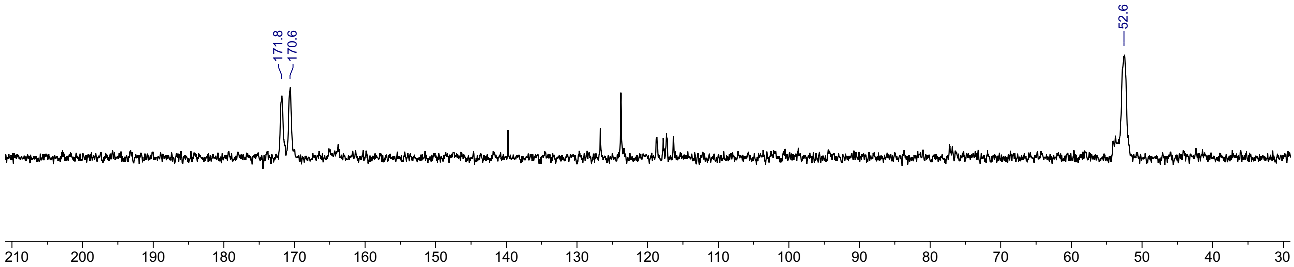

Figure S59. $^{31}P\{^1H\}$ NMR spectrum of $[Rh(JEKphos)(COEt)(CO)_2]$ (202 MHz, d_8-toluene).

3 References

[1] Pike, S. D.; Crimmin, M. R.; Chaplin, A. B. Organometallic Chemistry Using Partially Fluorinated Benzenes. *Chem. Commun.* **2017**, *53*, 3615–3633.

[2] (a) Castro, P. P.; Zhao, G.; Masangkay, G. A.; Hernandez, C.; Gutierrez-Tunstad, L. M. Quinoxaline Excision: A Novel Approach to Tri- and Diquinoxaline Cavitands. *Org. Lett.* **2004**, *6*, 333–336; (b) Dalcanale, E.; Soncini, P.; Bacchilega, G.; Ugozzoli, F. Selective Complexation of Neutral Molecules in Organic Solvents. Host–Guest Complexes and Cavitates between Cavitands and Aromatic Compounds. *J. Chem. Soc. Chem. Commun.* **1989**, 500–502.

[3] (a) Chaplin, A. B.; Hooper, J. F.; Weller, A. S.; Willis, M. C. Intermolecular Hydroacylation: High Activity Rhodium Catalysts Containing Small-Bite-Angle Diphosphine Ligands. *J. Am. Chem. Soc.* **2012**, *134*, 4885–4897; (b) Abel, E. W.; Bennett, M. A.; Wilkinson, G. Norbornadiene–Metal Complexes and Some Related Compounds. *J. Chem. Soc.* **1959**, 3178–3182.

[4] (a) Krossing, I. The Facile Preparation of Weakly Coordinating Anions: Structure and Characterisation of Silverpolyfluoroalkoxyaluminates $AgAl(OR_F)_4$, Calculation of the Alkoxide Ion Affinity. *Chem. Eur. J.* **2001**, *7*, 490–502; (b) Krossing, I.; Reisinger, A. Chemistry with Weakly-Coordinating Fluorinated Alkoxyaluminate Anions: Gas Phase Cations in Condensed Phases? *Coord. Chem. Rev.* **2006**, *250*, 2721–2744.

[5] (a) Martínez-Martínez, A. J.; Weller, A. S. Solvent-Free Anhydrous Li^+, Na^+ and K^+ Salts of $[B(3,5\text{-}(CF_3)_2C_6H_3)_4]^-$, $[BAr^F_4]^-$. Improved Synthesis and Solid-State Structures. *Dalton Trans.* **2019**, *48*, 3551–3554; (b) Buschmann, W. E.; Miller, J. S.; Bowman-James, K.; Miller, C. N. Synthesis of $[M^{II}(NCMe)_6]^{2+}$ (M = V, Cr, Mn, Fe, Co, Ni) Salts of Tetra[3,5-Bis(Trifluoromethyl)Phenyl]Borate. *Inorg. Synth.* **2002**, *33*, 83–91.

[6] (a) Reed, C. A. H^+, CH_3^+, and R_3Si^+ Carborane Reagents: When Triflates Fail. *Acc. Chem. Res.* **2010**, *43*, 121–128; (b) Stasko, D.; Reed, C. A. Optimizing the Least Nucleophilic Anion. A New, Strong Methyl$^+$ Reagent. *J. Am. Chem. Soc.* **2002**, *124*, 1148–114.

[7] Pregosin, P. S. *NMR in Organometallic Chemistry*, Wiley, **2012**, pp. 251–254.

[8] Sheldrick, G. M. SHELXT – Integrated Space-Group and Crystal-Structure Determination. *Acta Cryst.* **2015**, *71*, 3–8.

[9] Dolomanov, O. V.; Bourhis, L. J.; Gildea, R. J.; Howard, J. A. K.; Puschmann, H. OLEX2: A Complete Structure Solution, Refinement and Analysis Program. *J. Appl. Cryst.* **2009**, *42*, 339–341.

[10] Iwasawa, T.; Nishimoto, Y.; Hama, K.; Kamei, T.; Nishiuchi, M.; Kawamura, Y. Synthesis of the Functionalized Cavitands with Inwardly Directed Dialkylsilyl Groups and Phosphorous Lone Pairs. *Tetrahedron Lett.* **2008**, *49*, 4758–4762.

[11] Maglic, J. B.; Lavendomme, R. MoloVol: An Easy-to-Use Program for Analyzing Cavities, Volumes and Surface Areas of Chemical Structures. *J. Appl. Crystallogr.* **2022**, *55*, 1033–1044.

[12] Fennis, P. J.; Budzelaar, P. H. M.; Frijns, J. H. G.; Orpen, A. G. Dichloromethane Addition to Rhodium-*β*-Diketonate Complexes of Diphosphines and Pyridyl-Substituted Diphosphines. *J. Organomet. Chem.* **1990**, *393*, 287–298.
